# Supplementary material for: Impact of ligand binding on VEGFR1, VEGFR2, and NRP1 localization in human endothelial cells
Source: PLoS Comput Biol. 2025 Jul 16;21(7):e1013254. doi: 10.1371/journal.pcbi.1013254 (PMC12310042; doi:10.1371/journal.pcbi.1013254)
Supplement: S1 Table — While all four ligands bind VEGFR1, PLGF ligands do not bind VEGFR2. Longer isoforms include a NRP1-binding domain. VEGFR1 and NRP1 can directly interact, and the coupled receptor does not admit the NRP1-binding ligands, rather the non-NRP1-binding ligands can bind to the VEGFR1 in the complex. VEGFR2 and NRP1 do not directly interact, and only form a complex when bridged by a VEGF165a molecule binding to both receptors. (PDF) [file pcbi.1013254.s001.pdf]

**S1 Table. Ligand-receptor interactions.** While all four ligands bind VEGFR1, PLGF ligands do not bind VEGFR2. Longer isoforms include a NRP1-binding domain. VEGFR1 and NRP1 can directly interact, and the coupled receptor does not admit the NRP1-binding ligands, rather the non-NRP1-binding ligands can bind to the VEGFR1 in the complex. VEGFR2 and NRP1 do not directly interact, and only form a complex when bridged by a $VEGF_{165a}$ molecule binding to both receptors.

| | VEGFR1 | VEGFR2 | NRP1 | VEGFR1-NRP1 |
|---|---|---|---|---|
| **$VEGF_{121a}$** | X | X | | X |
| **$VEGF_{165a}$** | X | X | X | |
| **$PLGF_1$** | X | | | X |
| **$PLGF_2$** | X | | X | |
