# Supplementary material for: Impact of ligand binding on VEGFR1, VEGFR2, and NRP1 localization in human endothelial cells
Source: PLoS Comput Biol. 2025 Jul 16;21(7):e1013254. doi: 10.1371/journal.pcbi.1013254 (PMC12310042; doi:10.1371/journal.pcbi.1013254)
Supplement: S2 Table — The total number of molecules and molecular complexes in the model is 281. The comprehensive lists of these molecules, and the unique ID number by which each is identified in the code, are given in S3–S11 Tables. (PDF) [file pcbi.1013254.s002.pdf]

**S2 Table. Molecules included in the model.** The total number of molecules and molecular complexes in the model is 281. The comprehensive lists of these molecules, and the unique ID number by which each is identified in the code, are given in S3-S11 Tables.

| Type | Number of complexes | See Table |
|---|---|---|
| Unligated Receptors and Receptor Complexes | 32 | S3 |
| Unbound Ligands | 16 | S4 |
| Ligand-bound monomeric R1 or R2 | 32 | S5 |
| Nonsignaling ligand-bound VEGFR1 dimers | 48 | S6 |
| Nonsignaling ligand-bound VEGFR2 dimers | 24 | S7 |
| Nonsignaling NRP1-only complexes | 16 | S8 |
| Signaling ligand-bound VEGFR1 dimers | 64 | S9 |
| Signaling ligand-bound VEGFR2 dimers | 32 | S10 |
| Matrix-bound ligands and receptors | 17 | S11 |
