# Supplementary material for: Impact of ligand binding on VEGFR1, VEGFR2, and NRP1 localization in human endothelial cells
Source: PLoS Comput Biol. 2025 Jul 16;21(7):e1013254. doi: 10.1371/journal.pcbi.1013254 (PMC12310042; doi:10.1371/journal.pcbi.1013254)
Supplement: S3 Table — This table gives the unique ID number by which each molecule or molecular complex is identified in the model code. Dots indicate direct binding. These unligated receptors were included in our previous model of the trafficking of only receptors [41]. (PDF) [file pcbi.1013254.s003.pdf]

S3 Table. Unligated receptors and receptor complexes. This table gives the unique ID number by which each molecule or molecular complex is identified in the model code. Dots indicate direct binding. These unligated receptors were included in our previous model of the trafficking of only receptors [41].

**Molecule/Complex**	**Surface**	**Rab4a5a**	**Rab11a**	**Lysosome (degraded)**
R1	5	22	70	77
R2	6	23	71	78
N1	7	24	72	79
R1.R1	9	31	90	147
R2.R2	10	32	91	148
N1.R1	11	33	92	149
N1.R1.R1	29	88	160	206
N1.R1.R1.N1	30	89	161	207
