# Supplementary material for: Impact of ligand binding on VEGFR1, VEGFR2, and NRP1 localization in human endothelial cells
Source: PLoS Comput Biol. 2025 Jul 16;21(7):e1013254. doi: 10.1371/journal.pcbi.1013254 (PMC12310042; doi:10.1371/journal.pcbi.1013254)
Supplement: S4 Table — This table gives the unique ID number by which each molecule is identified in the model code. V165 represents VEGF165a, V121 represents VEGF121a, P1 represents PLGF1, and P2 represents PLGF2. (PDF) [file pcbi.1013254.s004.pdf]

S4 Table. Unbound ligands. This table gives the unique ID number by which each molecule is identified in the model code. V165 represents $VEGF_{165a}$, V121 represents $VEGF_{121a}$, P1 represents $PLGF_1$, and P2 represents $PLGF_2$.

**Molecule/Complex**	**Surface**	**Rab4a5a**	**Rab11a**	**Lysosome (degraded)**
V165	1	25	73	80
V121	2	28	76	83
P1	3	26	74	81
P2	4	27	75	82
