# Supplementary material for: Impact of ligand binding on VEGFR1, VEGFR2, and NRP1 localization in human endothelial cells
Source: PLoS Comput Biol. 2025 Jul 16;21(7):e1013254. doi: 10.1371/journal.pcbi.1013254 (PMC12310042; doi:10.1371/journal.pcbi.1013254)
Supplement: S5 Table — These molecular complexes are non-signaling and primarily are intermediates to ligands binding two receptors. Dots indicate direct binding. This table gives the unique ID number by which each molecule or molecular complex is identified in the model code. V165 represents VEGF165a, V121 represents VEGF121a, P1 represents PLGF1, and P2 represents PLGF2. (PDF) [file pcbi.1013254.s005.pdf]

S5 Table. Ligand-bound monomeric VEGFR1 or VEGFR2. These molecular complexes are non-signaling and primarily are intermediates to ligands binding two receptors. Dots indicate direct binding. This table gives the unique ID number by which each molecule or molecular complex is identified in the model code. V165 represents $VEGF_{165a}$, V121 represents $VEGF_{121a}$, P1 represents $PLGF_1$, and P2 represents $PLGF_2$.

Molecule/Complex	Surface	Rab4a5a	Rab11a	Lysosome (degraded)
V165.R1	18	52	109	153
V121.R1	19	53	110	154
V121.R1.N1	46	103	172	219
P1.R1	20	54	111	155
P1.R1.N1	47	104	173	224
P2.R1	21	55	112	157
V165.R2	16	50	107	151
V121.R2	17	51	108	152
