# Supplementary material for: Impact of ligand binding on VEGFR1, VEGFR2, and NRP1 localization in human endothelial cells
Source: PLoS Comput Biol. 2025 Jul 16;21(7):e1013254. doi: 10.1371/journal.pcbi.1013254 (PMC12310042; doi:10.1371/journal.pcbi.1013254)
Supplement: S6 Table — Although these complexes include two receptors, they are not coupled by the ligand (i.e., the ligand is bound only to one of the two receptors) and thus are not in higher-propensity signaling conformation. These are also likely to be intermediate forms. Dots and parentheses indicate direct binding; for example, P1.R1(N1).R1 means one PLGF1 is bound to one VEGFR1, and this VEGFR1 is itself bound to another VEGFR1 and to a NRP1. This table gives the unique ID number by which each molecule or molecular complex is identified in the model code. V165 represents VEGF165a, V121 represents VEGF121a, P1 represents PLGF1, and P2 represents PLGF2. (PDF) [file pcbi.1013254.s006.pdf]

S6 Table. Non-signaling ligand-bound VEGFR1 dimers. Although these complexes include two receptors, they are not coupled by the ligand (i.e. the ligand is bound only to one of the two receptors) and thus are not in higher-propensity signaling conformation. These are also likely to be intermediate forms. Dots and parentheses indicate direct binding; for example, P1.R1(N1).R1 means one $PLGF_1$ is bound to one VEGFR1, and this VEGFR1 is itself bound to another VEGFR1 and to a NRP1. This table gives the unique ID number by which each molecule or molecular complex is identified in the model code. V165 represents $VEGF_{165a}$, V121 represents $VEGF_{121a}$, P1 represents $PLGF_1$, and P2 represents $PLGF_2$.

**Molecule/Complex**	**Surface**	**Rab4a5a**	**Rab11a**	**Lysosome (degraded)**
V165.R1.R1	42	99	168	216
V165.R1.R1.N1	86	158	230	261
V121.R1.R1	43	100	169	218
V121.R1.R1.N1	66	143	202	220
V121.R1(N1).R1	93	162	232	263
V121.R1(N1).R1.N1	94	163	233	265
P1.R1.R1	44	101	170	223
P1.R1.R1.N1	68	145	204	225
P1.R1(N1).R1	95	164	234	268
P1.R1(N1).R1.N1	96	165	235	270
P2.R1.R1	45	102	171	229
P2.R1.R1.N1	87	159	231	273
