# Supplementary material for: Impact of ligand binding on VEGFR1, VEGFR2, and NRP1 localization in human endothelial cells
Source: PLoS Comput Biol. 2025 Jul 16;21(7):e1013254. doi: 10.1371/journal.pcbi.1013254 (PMC12310042; doi:10.1371/journal.pcbi.1013254)
Supplement: S7 Table — Although these complexes include two receptors, they are not coupled by the ligand (i.e., the ligand is bound only to one of the two receptors) and thus are not in higher-propensity signaling conformation. These are also likely to be intermediate forms. Dots and parentheses indicate direct binding. This table gives the unique ID number by which each molecule or molecular complex is identified in the model code. V165 represents VEGF165a and V121 represents VEGF121a. (PDF) [file pcbi.1013254.s007.pdf]

S7 Table. Non-signaling ligand-bound VEGFR2 dimers. Although these complexes include two receptors, they are not coupled by the ligand (i.e. the ligand is bound only to one of the two receptors) and thus are not in higher-propensity signaling conformation. These are also likely to be intermediate forms. Dots and parentheses indicate direct binding. This table gives the unique ID number by which each molecule or molecular complex is identified in the model code. V165 represents $VEGF_{165a}$ and V121 represents $VEGF_{121a}$.

**Molecule/Complex**	**Surface**	**Rab4a5a**	**Rab11a**	**Lysosome (degraded)**
N1.V165.R2	64	141	200	211
N1.V165(N1).R2	128	187	249	257
V165.R2.R2	40	97	166	210
N1.V165.R2.R2	65	142	201	212
N1.V165(N1).R2.R2	130	189	251	258
V121.R2.R2	41	98	167	213
