# Supplementary material for: Impact of ligand binding on VEGFR1, VEGFR2, and NRP1 localization in human endothelial cells
Source: PLoS Comput Biol. 2025 Jul 16;21(7):e1013254. doi: 10.1371/journal.pcbi.1013254 (PMC12310042; doi:10.1371/journal.pcbi.1013254)
Supplement: S8 Table — Dots indicate direct binding. This table gives the unique ID number by which each molecule or molecular complex is identified in the model code. V165 represents VEGF165a and P2 represents PLGF2. (PDF) [file pcbi.1013254.s008.pdf]

S8 Table. Nonsignaling NRP1-only complexes. Dots indicate direct binding. This table gives the unique ID number by which each molecule or molecular complex is identified in the model code. V165 represents $VEGF_{165a}$ and P2 represents $PLGF_2$.

**Molecule/Complex**	**Surface**	**Rab4a5a**	**Rab11a**	**Lysosome (degraded)**
V165.N1	14	48	105	150
P2.N1	15	49	106	156
N1.V165.N1	56	133	192	208
N1.P2.N1	57	134	193	227
