# Supplementary material for: Impact of ligand binding on VEGFR1, VEGFR2, and NRP1 localization in human endothelial cells
Source: PLoS Comput Biol. 2025 Jul 16;21(7):e1013254. doi: 10.1371/journal.pcbi.1013254 (PMC12310042; doi:10.1371/journal.pcbi.1013254)
Supplement: S9 Table — This table gives the unique ID number by which each molecule or molecular complex is identified in the model code. Dots indicate direct binding. A ∆ symbol indicates that the ligand is bound to both VEGFR1, and that the two VEGFR1 are also directly associated with each other. V165 represents VEGF165a, V121 represents VEGF121a, P1 represents PLGF1, and P2 represents PLGF2. (PDF) [file pcbi.1013254.s009.pdf]

S9 Table. Signaling ligand-bound VEGFR1 dimers. This table gives the unique ID number by which each molecule or molecular complex is identified in the model code. Dots indicate direct binding. A ∆ symbol indicates that the ligand is bound to both VEGFR1, and that the two VEGFR1 are also directly associated with each other. V165 represents $VEGF_{165a}$, V121 represents $VEGF_{121a}$, P1 represents $PLGF_1$, and P2 represents $PLGF_2$.

Molecule/Complex	Surface	Rab4a5a	Rab11a	Lysosome (degraded)
R1.V165.R1	60	137	196	215
R1.V121.R1	61	138	197	217
R1.V121.R1.N1	67	144	203	221
N1.R1.V121.R1.N1	131	190	252	266
R1.V165.R1 ∆	121	181	243	260
R1.V121.R1 ∆	122	182	244	262
R1.V121.R1.N1 ∆	123	183	245	264
N1.R1.V121.R1.N1 ∆	175	237	275	280
R1.P1.R1	62	139	198	222
R1.P1.R1.N1	69	146	205	226
N1.R1.P1.R1.N1	132	191	253	271
R1.P2.R1	63	140	199	228
R1.P1.R1 ∆	124	184	246	267
R1.P1.R1.N1 ∆	125	185	247	269
N1.R1.P1.R1.N1 ∆	176	238	276	281
R1.P2.R1 ∆	127	186	248	272
