# Supplementary material for: Impact of ligand binding on VEGFR1, VEGFR2, and NRP1 localization in human endothelial cells
Source: PLoS Comput Biol. 2025 Jul 16;21(7):e1013254. doi: 10.1371/journal.pcbi.1013254 (PMC12310042; doi:10.1371/journal.pcbi.1013254)
Supplement: S10 Table — This table gives the unique ID number by which each molecule or molecular complex is identified in the model code. Dots and parentheses indicate direct binding. A ∆ symbol indicates that the ligand is bound to both VEGFR2, and that the two VEGFR2 are also directly associated with each other. V165 represents VEGF165a and V121 represents VEGF121a. (PDF) [file pcbi.1013254.s010.pdf]

S10 Table. Signaling ligand-bound VEGFR2 dimers. This table gives the unique ID number by which each molecule or molecular complex is identified in the model code. Dots and parentheses indicate direct binding. A ∆ symbol indicates that the ligand is bound to both VEGFR2, and that the two VEGFR2 are also directly associated with each other. V165 represents $VEGF_{165a}$ and V121 represents $VEGF_{121a}$.

Molecule/Complex	Surface	Rab4a5a	Rab11a	Lysosome (degraded)
R2.V165.R2	58	135	194	209
R2.V165(N1).R2	129	188	250	255
R2.(N1)V165(N1).R2	177	239	277	278
R2.V121.R2	59	136	195	214
R2.V165.R2 ∆	117	178	240	254
R2.V165(N1).R2 ∆	118	179	241	256
R2.(N1)V165(N1).R2 ∆	174	236	274	279
R2.V121.R2 ∆	119	180	242	259
