# Supplementary material for: Impact of ligand binding on VEGFR1, VEGFR2, and NRP1 localization in human endothelial cells
Source: PLoS Comput Biol. 2025 Jul 16;21(7):e1013254. doi: 10.1371/journal.pcbi.1013254 (PMC12310042; doi:10.1371/journal.pcbi.1013254)
Supplement: S11 Table — Due to the size of matrix molecules, these complexes are only at the surface and are not internalized. However, ligand-coupled receptors can still serve as signal initiation [53]. This table gives the unique ID number by which each molecule or molecular complex is identified in the model code. Dots and parentheses indicate direct binding. A ∆ symbol indicates that the ligand is bound to both VEGFR, and that the two VEGFR are also associated. This table is included for complete description of the code provided, however in the present study matrix binding is not included and all these molecules will have zero concentration. V165 represents VEGF165a, V121 represents VEGF121a, P1 represents PLGF1, and P2 represents PLGF2. (PDF) [file pcbi.1013254.s011.pdf]

**S11 Table. Matrix-bound complexes.** Due to the size of matrix molecules, these complexes are only at the surface and are not internalized. However, ligand-coupled receptors can still serve as signal initiation [53]. This table gives the unique ID number by which each molecule or molecular complex is identified in the model code. Dots and parentheses indicate direct binding. A $\Delta$ symbol indicates that the ligand is bound to both VEGFR, and that the two VEGFR are also associated. This table is included for complete description of the code provided, however in the present study matrix binding is not included and all these molecules will have zero concentration. V165 represents $VEGF_{165a}$, V121 represents $VEGF_{121a}$, P1 represents $PLGF_1$, and P2 represents $PLGF_2$.

| Molecule/Complex | Surface | Signaling |
|---|---|---|
| M | 8 | |
| M.V165 | 12 | |
| M.V165.R1 | 34 | |
| M.V165.R1.R1 | 37 | |
| M.V165.R1.R1.N1 | 84 | |
| R1.(M)V165.R1 | 114 | Yes |
| R1.(M)V165.R1 $\Delta$ | 120 | Yes |
| M.V165.R2 | 36 | |
| M.V165.R2.R2 | 39 | |
| R2.(M)V165.R2 | 113 | Yes |
| R2.(M)V165.R2 $\Delta$ | 116 | Yes |
| M.P2 | 13 | |
| M.P2.R1 | 35 | |
| M.P2.R1.R1 | 38 | |
| M.P2.R1.R1.N1 | 85 | |
| R1.(M)P2.R1 | 115 | Yes |
| R1.(M)P2.R1 $\Delta$ | 126 | Yes |
