# Supplementary material for: Impact of ligand binding on VEGFR1, VEGFR2, and NRP1 localization in human endothelial cells
Source: PLoS Comput Biol. 2025 Jul 16;21(7):e1013254. doi: 10.1371/journal.pcbi.1013254 (PMC12310042; doi:10.1371/journal.pcbi.1013254)
Supplement: S12 Table — Compartment volumes, compartment surface areas, and initial concentrations of ligands and surface receptors. Ligand molecular weights were obtained from sources of experimental recombinant proteins. Includes sources for justification of key parameters from previous studies [41,74,75]. (PDF) [file pcbi.1013254.s012.pdf]

S12 Table. Model Parameters. Compartment volumes, compartment surface areas, and initial concentrations of ligands and surface receptors. Ligand molecular weights were obtained from sources of experimental recombinant proteins. Includes sources for justification of key parameters from previous studies [41,74,75].

Receptor species/ parameters	Value	Units	Reference or assumptions (MW: molecular weight)
Extracellular volume	10^{-8}	liter/cell	1 ml cell culture media in one well with 10^5 cells
Rab4a volume	11.25×10^{-15}	liter/cell	[41]
Rab11a volume	3.75×10^{-15}	liter/cell	[41]
Cell surface area	1,000	μm^2/cell	[74]
Rab4a surface area	950	μm^2/cell	[41]
Rab11a surface area	350	μm^2/cell	[41]
VEGFR1 (cell surface)	1,800	receptors per cell	[75]
VEGFR2 (cell surface)	4,900	receptors per cell	[75]
NRP1 (cell surface)	68,000	receptors per cell	[75]
$VEGF_{121a}$	1.075×10^7	molecules/cell	50 ng.mL^{-1} , MW ~28 kDa
$VEGF_{165a}$	6.843×10^6	molecules/cell	50 ng.mL^{-1} , MW ~44 kDa
$PLGF_1$	1.014×10^7	molecules/cell	50 ng.mL^{-1}, MW ~29.7 kDa
$PLGF_2$	8.702×10^6	molecules/cell	50 ng.mL^{-1}, MW ~34.6 kDa
