# Supplementary material for: Impact of ligand binding on VEGFR1, VEGFR2, and NRP1 localization in human endothelial cells
Source: PLoS Comput Biol. 2025 Jul 16;21(7):e1013254. doi: 10.1371/journal.pcbi.1013254 (PMC12310042; doi:10.1371/journal.pcbi.1013254)
Supplement: S13 Table — These parameters are obtained from optimization in the absence of ligands, and result in steady state surface receptor densities in agreement with previous measurements [41], when used in concert with the trafficking parameters in S14 Table. (PDF) [file pcbi.1013254.s013.pdf]

S13 Table. Production rates for VEGF receptors. These parameters are obtained from optimization in the absence of ligands, and result in steady state surface receptor densities in agreement with previous measurements [41], when used in concert with the trafficking parameters in S14 Table.

Production k_{prod}	VEGFR1	4.101	$(\#/cell).s^{-1}$
	VEGFR2	1.114	$(\#/cell).s^{-1}$
	NRP1	0.459	$(\#/cell).s^{-1}$
