# Supplementary material for: Impact of ligand binding on VEGFR1, VEGFR2, and NRP1 localization in human endothelial cells
Source: PLoS Comput Biol. 2025 Jul 16;21(7):e1013254. doi: 10.1371/journal.pcbi.1013254 (PMC12310042; doi:10.1371/journal.pcbi.1013254)
Supplement: S14 Table — This standard parameter set was used for model simulations presented in the main manuscript. Based on modeling and data from HUVECs (Human Umbilical Vein Endothelial Cells). Unligated VEGFR1, VEGFR2, and NRP1 trafficking parameters are from previous work [41]; ligated trafficking parameters are described in this study. R1: VEGFR1, R2: VEGFR2, N1: Neuropilin-1 or NRP1. (PDF) [file pcbi.1013254.s014.pdf]

S14 Table. Trafficking parameter estimates for VEGFR1, VEGFR2, and NRP1 in the absence and presence of ligands based on experimental data from HUVEC. This standard parameter set was used for model simulations presented in the main manuscript. Based on modeling and data from HUVECs (Human Umbilical Vein Endothelial Cells). Unligated VEGFR1, VEGFR2, and NRP1 trafficking parameters are from previous work [41]; ligated trafficking parameters are described in this study. R1: VEGFR1, R2: VEGFR2, N1: Neuropilin-1 or NRP1.

	**Receptor**	**Unliganded** ***k*** **(s^{-1})**	**VEGF-bound** ***k*** **(s^{-1})**	**PLGF-bound** ***k*** **(s^{-1})**
Internalization k_{int}	VEGFR1	1.3×10^{-2}	1.3×10^{-2}	1.3×10^{-2}
	VEGFR2	2.3×10^{-4}	$3 \times 2.3 \times 10^{-4}$	
	NRP1	2.7×10^{-4}	2.7×10^{-4}	2.7×10^{-4}
	R1.N1	1.3×10^{-2}	1.3×10^{-2}	1.3×10^{-2}
	R2.N1		$3 \times 2.3 \times 10^{-4}$	
Recycling to surface via Rab4a endosomes k_{rec4}	VEGFR1	5.4×10^{-4}	5.4×10^{-4}	5.4×10^{-4}
	VEGFR2	1.2×10^{-6}	1.2×10^{-6}	
	NRP1	2.1×10^{-2}	2.1×10^{-2}	2.1×10^{-2}
	R1.N1	5.4×10^{-4}	5.4×10^{-4}	5.4×10^{-4}
	R2.N1		1.2×10^{-6}	
Transfer from Rab4a endosomes to Rab11a endosomes k_{4to11}	VEGFR1	5.9×10^{-4}	5.9×10^{-4}	5.9×10^{-4}
	VEGFR2	1.5×10^{-6}	1.5×10^{-6}	
	NRP1	7.0×10^{-2}	7.0×10^{-2}	7.0×10^{-2}
	R1.N1	5.9×10^{-4}	5.9×10^{-4}	5.9×10^{-4}
	R2.N1		1.5×10^{-6}	
Recycling to surface via Rab11a endosomes k_{rec11}	VEGFR1	1.0×10^{-1}	1.0×10^{-1}	1.0×10^{-1}
	VEGFR2	8.9×10^{-2}	8.9×10^{-2}	
	NRP1	7.9×10^{-4}	7.9×10^{-4}	7.9×10^{-4}
	R1.N1	1.0×10^{-1}	1.0×10^{-1}	1.0×10^{-1}
	R2.N1		8.9×10^{-2}	
Degradation k_{deg}	VEGFR1	2.3×10^{-4}	2.3×10^{-4}	2.3×10^{-4}
	VEGFR2	2.3×10^{-4}	2.3×10^{-4}	
	NRP1	1.2×10^{-6}	1.2×10^{-6}	1.2×10^{-6}
	R1.N1	2.3×10^{-4}	2.3×10^{-4}	2.3×10^{-4}
	R2.N1		2.3×10^{-4}	
