# Supplementary material for: Impact of ligand binding on VEGFR1, VEGFR2, and NRP1 localization in human endothelial cells
Source: PLoS Comput Biol. 2025 Jul 16;21(7):e1013254. doi: 10.1371/journal.pcbi.1013254 (PMC12310042; doi:10.1371/journal.pcbi.1013254)
Supplement: S15 Table — R1: VEGFR1, R2: VEGFR2, N1: Neuropilin-1/NRP1. The unligated receptor dimerization rates were set in a previous study to yield 30–40% dimers of R1-R1, R2-R2, and N1-R1 [41]. Note that these base parameters (in units of molecules-1.µm2.s-1) are adjusted to units of molecules-1.cell.s-1 at each location, using the appropriate membrane surface area (S12 Table), as described previously [41]. Includes sources for justification of key parameters from previous studies [7,8,41,45,53,76]. (PDF) [file pcbi.1013254.s015.pdf]

S15 Table. Receptor dimerization parameters. R1: VEGFR1, R2: VEGFR2, N1: Neuropilin-1/NRP1. The unligated receptor dimerization rates were set in a previous study to yield 30-40% dimers of R1-R1, R2-R2, and N1-R1 [41]. Note that these base parameters (in units of molecules^{-1}.µm^{2}.s^{-1}) are adjusted to units of molecules^{-1}.cell.s^{-1} at each location, using the appropriate membrane surface area (S12 Table), as described previously [41]. Includes sources for justification of key parameters from previous studies [7,8,41,45,53,76].

	**Description**	**$k_{on,RR}$ (molecules^{-1}.µm^{2}.s^{-1})**	**k_{off} (s^{-1})**	**K_D (molecules. µm^{-2})**	**Reference**
R1-R1	unligated VEGFR1 dimerization	8.0×10^{-4}	1.0×10^{-2}	12.5	See [41]
R2-R2	unligated VEGFR2 dimerization	2.0×10^{-3}	1.0×10^{-2}	5	[7,8] & see [41]
N1-R1	unligated NRP1-VEGFR1 dimerization	8.0×10^{-4}	1.0×10^{-2}	12.5	[41,45,53,76]

	**Description**	**$k_{on,RR}$ Surface (molecules^{-1}.cell.s^{-1})**	**k_{on} Rab4a (molecules^{-1}.cell.s^{-1})**	**k_{on} Rab11a (molecules^{-1}.cell.s^{-1})**	**Reference**
R1-R1	unligated VEGFR1 dimerization	8.0×10^{-7}	8.42×10^{-7}	2.46×10^{-6}	See [41]
R2-R2	unligated VEGFR2 dimerization	2.0×10^{-6}	2.11×10^{-6}	6.15×10^{-6}	See [41]
N1-R1	unligated NRP1-VEGFR1 dimerization	8.0×10^{-7}	8.42×10^{-7}	2.46×10^{-6}	See [41]
