# Supplementary material for: Impact of ligand binding on VEGFR1, VEGFR2, and NRP1 localization in human endothelial cells
Source: PLoS Comput Biol. 2025 Jul 16;21(7):e1013254. doi: 10.1371/journal.pcbi.1013254 (PMC12310042; doi:10.1371/journal.pcbi.1013254)
Supplement: S16 Table — Measured and estimated rate constants and equilibrium constants assuming 1:1 interaction (i.e., monovalent ligand binds monovalent receptor dimer fully in one step; this is the most common assumption for estimating ligand-receptor binding experimentally). These are not the values used in our model, which is a dimerization-explicit model, but the parameters used (S17 Table) are based on these as described in the Methods section. In each entry the units of the parameters shown are (top to bottom): KD (pM), kon (pM-1 s-1), and koff (s-1). L: Ligand; R1: VEGFR1; R2: VEGFR2; N1: NRP1. Includes sources for justification of key parameters from previous studies [34,53,76–79]. (PDF) [file pcbi.1013254.s016.pdf]

S16 Table. Experimentally-derived 1:1 Ligand-Receptor binding. Measured and estimated rate constants and equilibrium constants assuming 1:1 interaction (i.e. monovalent ligand binds monovalent receptor dimer fully in one step; this is the most common assumption for estimating ligand-receptor binding experimentally). These are not the values used in our model, which is a dimerization-explicit model, but the parameters used (S17 Table) are based on these as described in the Methods section. In each entry the units of the parameters shown are (top to bottom): K_D (pM), k_{on} ($pM^{-1}\ s^{-1}$), and k_{off} (s^{-1}). L: Ligand; R1: VEGFR1; R2: VEGFR2; N1: NRP1. Includes sources for justification of key parameters from previous studies [34,53,76,77,78,79].

Interaction	Parameter	$VEGF_{121a}$	$VEGF_{165a}$	$PLGF_1$	$PLGF_2$	Reference
L-R1	K_d<br>k_{on}<br>k_{off}	33<br>3×10^{-5}<br>10^{-3}	33<br>3×10^{-5}<br>10^{-3}	233<br>1.5×10^{-6}<br>3.5×10^{-4}	233<br>1.5×10^{-6}<br>3.5×10^{-4}	[53,76]
L-R2	K_d<br>k_{on}<br>k_{off}	100<br>1×10^{-5}<br>10^{-3}	100<br>1×10^{-5}<br>10^{-3}			[34,79,76]
L-N1	K_d<br>k_{on}<br>k_{off}		1,200<br>5×10^{-7}<br>6×10^{-4}		100,000<br>1×10^{-8}<br>1×10^{-3}	[77,78,79]
L-(N1R1)	K_d<br>k_{on}<br>k_{off}	33<br>3×10^{-5}<br>1×10^{-3}		233<br>1.5×10^{-6}<br>3.5×10^{-4}		[77,78,79]
