# Supplementary material for: Impact of ligand binding on VEGFR1, VEGFR2, and NRP1 localization in human endothelial cells
Source: PLoS Comput Biol. 2025 Jul 16;21(7):e1013254. doi: 10.1371/journal.pcbi.1013254 (PMC12310042; doi:10.1371/journal.pcbi.1013254)
Supplement: S17 Table — Calculated rate constants for first binding step to monomers or dimers assuming 1:2 interaction (i.e., one bivalent ligand can bind to two monovalent receptor monomers, or twice to one bivalent receptor dimer). The base on-rate constants (kon) are assumed to be one quarter of the 1:1 rate constant (S16 Table) to account for the bivalency of ligands and of receptor dimers. Thus, when considering these base on-rate constant values below, ligand binding to a receptor monomer is twice the rate constant (due to two receptor-binding sites on the ligand) and ligand binding to an unliganded receptor dimer is four times the rate constant (due to two receptor-binding sites on the ligands and two ligand-binding sites on the receptor dimer). L: Ligand; R1: VEGFR1; R2: VEGFR2; N1: NRP1. (PDF) [file pcbi.1013254.s017.pdf]

S17 Table. Ligand-Receptor binding. Calculated rate constants for first binding step to monomers or dimers assuming 1:2 interaction (i.e. one bivalent ligand can bind to two monovalent receptor monomers, or twice to one bivalent receptor dimer). The base on-rate constants (k_{on}) are assumed to be one quarter of the 1:1 rate constant (S16 Table) to account for the bivalency of ligands and of receptor dimers. Thus, when considering these base on-rate constant values below, ligand binding to a receptor monomer is twice the rate constant (due to two receptor-binding sites on the ligand) and ligand binding to an unliganded receptor dimer is four times the rate constant (due to two receptor-binding sites on the ligands and two ligand-binding sites on the receptor dimer). L: Ligand; R1: VEGFR1; R2: VEGFR2; N1: NRP1.

Interaction	Rate Constant	$VEGF_{121a}$	$VEGF_{165a}$	$PLGF_1$	$PLGF_2$
L-R1	k_{on} (pM^{-1} s^{-1})	2* or 4*<br>7.5×10^{-6}	2* or 4*<br>7.5×10^{-6}	2* or 4*<br>3.75×10^{-7}	2* or 4*<br>3.75×10^{-7}
	k_{on} $(\#/cell)^{-1}s^{-1}$	2* or 4*	2* or 4*	2* or 4*	2* or 4*
	surface	1.25×10^{-9}	1.25×10^{-9}	6.23×10^{-11}	6.23×10^{-11}
	rab4a	1.11×10^{-3}	1.11×10^{-3}	5.54×10^{-5}	5.54×10^{-5}
	rab11a	3.32×10^{-3}	3.32×10^{-3}	1.66×10^{-4}	1.66×10^{-4}
	k_{off} (s^{-1})	2.24×10^{-2}	2.24×10^{-2}	1.32×10^{-2}	1.32×10^{-2}
L-R2	k_{on} (pM^{-1} s^{-1})	2* or 4*<br>2.5×10^{-6}	2* or 4*<br>2.5×10^{-6}		
	k_{on} $(\#/cell)^{-1}s^{-1}$	2* or 4*	2* or 4*		
	surface	4.15×10^{-10}	4.15×10^{-10}		
	rab4a	3.69×10^{-4}	3.69×10^{-4}		
	rab11a	1.11×10^{-3}	1.11×10^{-3}		
	k_{off} (s^{-1})	2.24×10^{-2}	2.24×10^{-2}		
L-N1	k_{on} (pM^{-1} s^{-1})		2*<br>1.25×10^{-7}		2*<br>2.5×10^{-9}
	k_{on} $(\#/cell)^{-1}s^{-1}$		2*		2*
	surface		2.08×10^{-11}		4.15×10^{-13}
	rab4a		1.85×10^{-5}		3.69×10^{-7}
	rab11a		5.54×10^{-5}		1.11×10^{-6}
	k_{off} (s^{-1})		1.73×10^{-2}		2.24×10^{-2}
L-(N1R1)	k_{on} (pM^{-1} s^{-1})	2* or 4*<br>7.5×10^{-6}		2* or 4*<br>3.75×10^{-7}	
	k_{on} $(\#/cell)^{-1}s^{-1}$	2* or 4*		2* or 4*	
	surface	1.25×10^{-9}		6.23×10^{-11}	
	rab4a	1.11×10^{-3}		5.54×10^{-5}	
	rab11a	3.32×10^{-3}		1.66×10^{-4}	
	k_{off} (s^{-1})	2.24×10^{-2}		1.32×10^{-2}	
