# Supplementary material for: Impact of ligand binding on VEGFR1, VEGFR2, and NRP1 localization in human endothelial cells
Source: PLoS Comput Biol. 2025 Jul 16;21(7):e1013254. doi: 10.1371/journal.pcbi.1013254 (PMC12310042; doi:10.1371/journal.pcbi.1013254)
Supplement: S18 Table — These are the values for intermolecular and intramolecular binding of ligands and receptors following the first ligand-receptor binding event. Values for receptor-receptor coupling without a ligand (kon,RR) are given in S15 Table. The binding rate constant for a ligand (already bound to a receptor monomer) binding to a second receptor monomer (kon,LR); the binding rate constant for a ligand (already bound to one receptor in a dimer) binding to the second receptor in a dimer (k∆,LR); and the binding rate constant for a receptor binding to the second receptor of a dimer (when both are already bound to a ligand) (k∆,RR) are calculated based on other known parameters as described in Methods and in a previous publication [48]. L: Ligand; R1: VEGFR1; R2: VEGFR2; N1: NRP1. (PDF) [file pcbi.1013254.s018.pdf]

S18 Table. Ligand-Receptor Coupling. These are the values for intermolecular and intramolecular binding of ligands and receptors following the first ligand-receptor binding event. Values for receptor-receptor coupling without a ligand ($k_{on,RR}$) are given in S15 Table. The binding rate constant for a ligand (already bound to a receptor monomer) binding to a second receptor monomer ($k_{on,LR}$); the binding rate constant for a ligand (already bound to one receptor in a dimer) binding to the second receptor in a dimer ($k_{\Delta,LR}$); and the binding rate constant for a receptor binding to the second receptor of a dimer (when both are already bound to a ligand) ($k_{\Delta,RR}$) are calculated based on other known parameters as described in *Methods* and in a previous publication [48]. L: Ligand; R1: VEGFR1; R2: VEGFR2; N1: NRP1.

Interaction	Rate Constant	$VEGF_{121a}$	$VEGF_{165a}$	$PLGF_1$	$PLGF_2$
L-R1	$k_{on,LR}$ (#/cell)$^{-1}$s^{-1}				
	surface	5.31×10^{-4}	5.31×10^{-4}	5.41×10^{-4}	5.41×10^{-4}
	rab4a	5.59×10^{-4}	5.59×10^{-4}	5.69×10^{-4}	5.69×10^{-4}
	rab11a	1.63×10^{-3}	1.63×10^{-3}	1.66×10^{-3}	1.66×10^{-3}
	$k_{on,RR}/k_{on,LR}$	1.51×10^{-3}	1.51×10^{-3}	1.48×10^{-3}	1.48×10^{-3}
	$k_{\Delta,LR}$ (s^{-1})	9.55×10^{-1}	9.55×10^{-1}	9.74×10^{-1}	9.74×10^{-1}
	$k_{\Delta,RR}$ (s^{-1})	1.44×10^{-3}	1.44×10^{-3}	1.44×10^{-3}	1.44×10^{-3}
L-R2	$k_{on,LR}$ (#/cell)$^{-1}$s^{-1}				
	surface	1.91×10^{-4}	1.91×10^{-4}		
	rab4a	2.01×10^{-4}	2.01×10^{-4}		
	rab11a	5.88×10^{-4}	5.88×10^{-4}		
	$k_{on,RR}/k_{on,LR}$	1.05×10^{-2}	1.05×10^{-2}		
	$k_{\Delta,LR}$ (s^{-1})	9.55×10^{-1}	9.55×10^{-1}		
	$k_{\Delta,RR}$ (s^{-1})	1.00×10^{-2}	1.00×10^{-2}		
L-N1	$k_{on,LR}$ (#/cell)$^{-1}$s^{-1}				
	surface		1.42×10^{-5}		1.41×10^{-5}
	rab4a		1.49×10^{-5}		1.48×10^{-5}
	rab11a		4.37×10^{-5}		4.32×10^{-5}
L-(N1R1)	$k_{on,LR}$ (#/cell)$^{-1}$s^{-1}				
	surface	5.31×10^{-4}		5.41×10^{-4}	
	rab4a	5.59×10^{-4}		5.69×10^{-4}	
	rab11a	1.63×10^{-3}		1.66×10^{-3}	
	$k_{on,RR}/k_{on,LR}$	1.51×10^{-3}		1.48×10^{-3}	
	$k_{\Delta,LR}$ (s^{-1})	9.55×10^{-1}		9.74×10^{-1}	
	$k_{\Delta,RR}$ (s^{-1})	1.44×10^{-3}		1.44×10^{-3}	
R2-L-N1	$k_{on,RLN}$ (#/cell)$^{-1}$s^{-1}		2.50×10^{-6}		
	surface		2.63×10^{-6}		
	rab4a		7.69×10^{-6}		
	rab11a				
