# Supplementary material for: Impact of ligand binding on VEGFR1, VEGFR2, and NRP1 localization in human endothelial cells
Source: PLoS Comput Biol. 2025 Jul 16;21(7):e1013254. doi: 10.1371/journal.pcbi.1013254 (PMC12310042; doi:10.1371/journal.pcbi.1013254)
Supplement: S19 Table — (PDF) [file pcbi.1013254.s019.pdf]

S19 Table. List of experimental reagents and antibodies.

**Reagents**	**Company (catalog #)**	
HUVECs	Lonza (#2519A)<br>Lot #s: 0000704189 and 0000661173	
HUVEC culture media and supplements	EBM-2 medium supplemented with the bullet kit (EGM-2) (Lonza)	
siRNA Rab4a oligonucleotide	ThermoFisher Scientific 439084 (s11675)	
siRNA Rab11a oligonucleotide	ThermoFisher Scientific 4390824 (s16702)<br>or Santa Cruz Biotechnology (sc3630)	
siRNA transfection reagent Lipofectamine 3000	Thermo Fisher Scientific (L3000001)	
Biotinylation kit	Pierce Cell Surface Biotinylation<br>and Protein Isolation Kit (#A44390)	
Recombinant Human PLGF$_1$	R&D (264-PGB-010)	
VEGF$_{165a}$	Genscript (Z02689)	
Antibody	Company	IB titer
VEGFR1 (membrane-integral)	CST (#2893)	1:1000
VEGFR2 (membrane-integral)	CST (#2479)	1:10000
NRP1 (membrane-integral)	R&D (AF3870)	1:1000
PECAM1	CST (#3528)	1:100000
α-Tubulin	CST (#3873)	1:100000
β-Actin	CST (#3700)	1:10000
Rab4a	ThermoFisher (MA5-17161)	1:1000
Rab11a	Abcam (ab65200), BD Bio (610656)	1:2000

Anti-mouse IgG, HRP-linked (secondary)	ThermoFisher (A16011)	1:10000
Anti-rabbit IgG, HRP-linked (secondary)	ThermoFisher (A16035)	1:10000

Abbreviations
IB: Immunoblot, **CST:** Cell Signaling Technologies
HUVECs: Human Umbilical Vein Endothelial Cells
VEGFR: Vascular Endothelial Growth Factor Receptor; **NRP:** Neuropilin
PECAM: Platelet Endothelial Cell Adhesion Molecule, also known as CD31
