# Supplementary material for: Impact of ligand binding on VEGFR1, VEGFR2, and NRP1 localization in human endothelial cells
Source: PLoS Comput Biol. 2025 Jul 16;21(7):e1013254. doi: 10.1371/journal.pcbi.1013254 (PMC12310042; doi:10.1371/journal.pcbi.1013254)
Supplement: S1 Text — (PDF) [file pcbi.1013254.s020.pdf]

S1 Text. Reconstructing equations from the provided tables and the model code.

To use the tables and code to reconstruct the equations, here is an example. For the complex R2.V121.R2_surf, i.e. VEGF121a bound to two VEGFR2 on the cell surface, we consult the supplemental tables to identify the unique ID number for that molecule; in **S10 Table**, we find that it is molecule 59.

Next consulting the model code (*All_Lig_Traff_model_Bionetgen_20231016.m*), the function "*calc_species_deriv*" gives this for equation 59, which is the ordinary differential equation describing the rate of change of the concentration of molecule 59:

```
Dspecies(59) = ratelaws(77) -ratelaws(212) -ratelaws(244) -ratelaws(318)
               +ratelaws(464) +ratelaws(626) +ratelaws(923);
```

For each of these rate terms, we consult the function "*calc_ratelaws*"; for example:

```
ratelaws(77) = expressions(63)*species(17)*species(6);
```

Here, expressions(63) is the parameter (rate constant) listed 63rd in *paramlist.txt*, which is kR2vegfA_R2on_surf, i.e. the on-rate of binding of a VEGFR2 monomer binding to the second VEGFR2 binding site on a V121 molecule that already has one VEGFR2 bound.

species(17) and species(6) are the concentrations of the molecules given ID numbers 17 and 6; again we consult the supplemental tables:

Table S3 -> 6 = VEGFR2 on the surface
Table S5 -> 17 = V121-VEGFR2 on the surface

so the first term is: + kR2vegfA_R2on_surf * [R2surf] * [V121.R2surf]

We can continue with the other rate terms to assemble the full equation.

From the function "*calc_ratelaws*":

```
ratelaws(212) = 2*expressions(48)*species(59);
ratelaws(244) = expressions(84)*species(59);
ratelaws(318) = expressions(112)*species(59);
ratelaws(464) = expressions(16)*species(119);
ratelaws(626) = expressions(127)*species(136);
ratelaws(923) = expressions(157)*species(195);
```

From the supplemental tables:

119 = R2.V121.R2_del_surf
136 = R2.V121.R2_rab45
195 = R2.V121.R2_rab11

and so the full equation is:

```
d[R2.V121.R2_surf]/dt =
  + kR2vegfA_R2on_surf * [R2surf] * [V121.R2surf]
  - 2 * kvegfA_R2off * [R2.V121.R2_surf]
  - kdeltaR2R2 * [R2.V121.R2_surf]
  - kVegfR2Rab5a * [R2.V121.R2_surf]
  + kR2R2off * [R2.V121.R2_del_surf]
  + kVegfR2Rab4a * [R2.V121.R2_rab45]
  + kVegfR2Rab11a * [R2.V121.R2_rab11]
```

Other equations can be recreated in a similar manner.
