# Supplementary material for: Impact of ligand binding on VEGFR1, VEGFR2, and NRP1 localization in human endothelial cells
Source: PLoS Comput Biol. 2025 Jul 16;21(7):e1013254. doi: 10.1371/journal.pcbi.1013254 (PMC12310042; doi:10.1371/journal.pcbi.1013254)
Supplement: S1 Fig — The system was simulated under three different assumptions: one-to-one (“121”) ligand receptor binding, i.e., pre-dimerization of receptors and single step ligand binding/activation; ligand-induced dimerization (“LID”), i.e., no receptor pre-dimerization; and a full dimerization model including all dimerization paths (“All”). The similarity between the “121” and “All” lines indicates that the dimerization model represents the observed equilibrium data well. Top row: VEGFR1 expression only; bottom row: VEGFR2 expression only. (PDF) [file pcbi.1013254.s021.pdf]

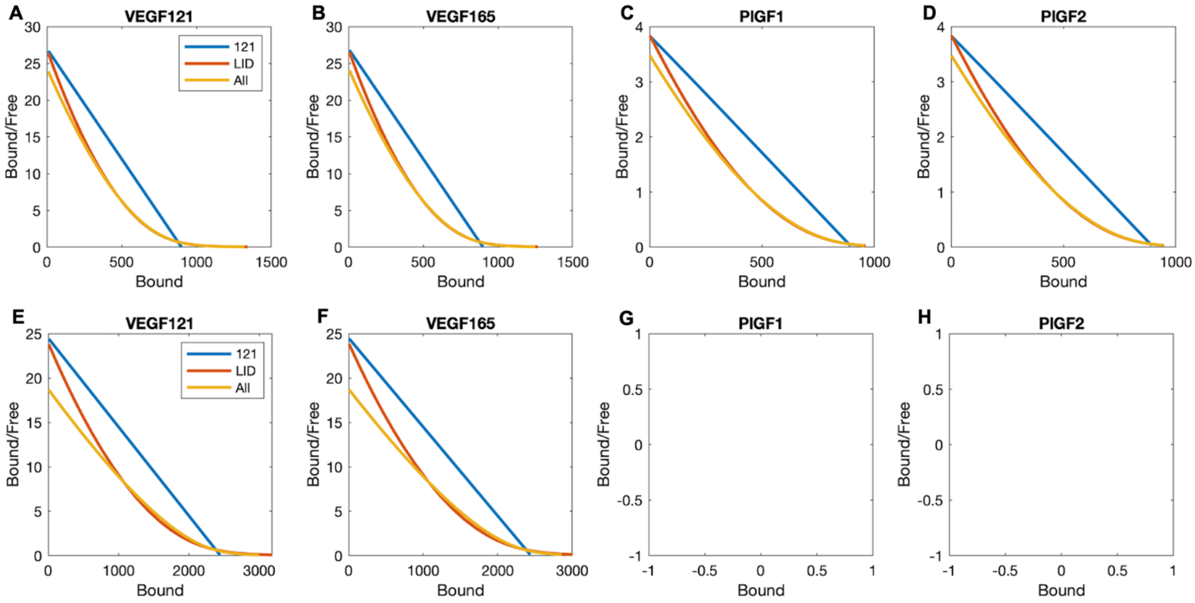

S1 Fig. Simulated Scatchard plots comparing different representations of VEGFR dimerization. The system was simulated under three different assumptions: one-to-one ("121") ligand receptor binding, i.e. pre-dimerization of receptors and single step ligand binding/activation; ligand-induced dimerization ("LID"), i.e. no receptor pre-dimerization; and a full dimerization model including all dimerization paths ("All"). The similarity between the "121" and "All" lines indicates that the dimerization model represents the observed equilibrium data well. Top row: VEGFR1 expression only; bottom row: VEGFR2 expression only.
