# Supplementary material for: Impact of ligand binding on VEGFR1, VEGFR2, and NRP1 localization in human endothelial cells
Source: PLoS Comput Biol. 2025 Jul 16;21(7):e1013254. doi: 10.1371/journal.pcbi.1013254 (PMC12310042; doi:10.1371/journal.pcbi.1013254)
Supplement: S2 Fig — Total (ligated and unligated) receptor levels on the cell surface, inside the cell, and across the whole cell in response to VEGF165a treatment at different levels of how the ligand binding affects the indicated VEGFR2 trafficking parameter. Simulations are shown as solid and dotted lines. Gray arrows indicate the direction of increasing parameter values. The same experimental data is shown in each row as dots and variance bars. A-C, VEGF165a binding causes an increase in the internalization rate constant for VEGFR2 (V.R2.kint and V.R2.N1.kint), compared to the unligated value; a three-fold increase (solid line) compared to the unligated receptor rate constant matched the observed data best. D-O, Taking this increased kint (receptor complex internalization rate) as a baseline for the remaining simulations, we explored variation in the other trafficking parameters. Lines represent 5x, 2x, 1x, 0.5x, 0.2x the baseline (unligated) trafficking rate; in panels D and G-O the lines overlap substantially. D-F, total surface, internal and whole cell receptors in response to VEGF165a treatment and change in ligated VEGFR2 degradation (altered kdeg affecting both V.R2 and V.R2.N1 complexes). G-O, total surface, internal and whole cell receptors in response to VEGF165a treatment and change in ligated VEGFR2 recycling, including: recycling via Rab4a (altered krec4, G-I); transfer to Rab11a (altered k4to11, J-L); and recycling via Rab11a (altered krec11, M-O). Altered rate constants affect both V.R2 and V.R2.N1 complexes. Panels A-F are identical to panels A-F of Fig 3; they are replicated here for ease of comparison. Gray arrows indicate the direction of increasing parameter values. (PDF) [file pcbi.1013254.s022.pdf]

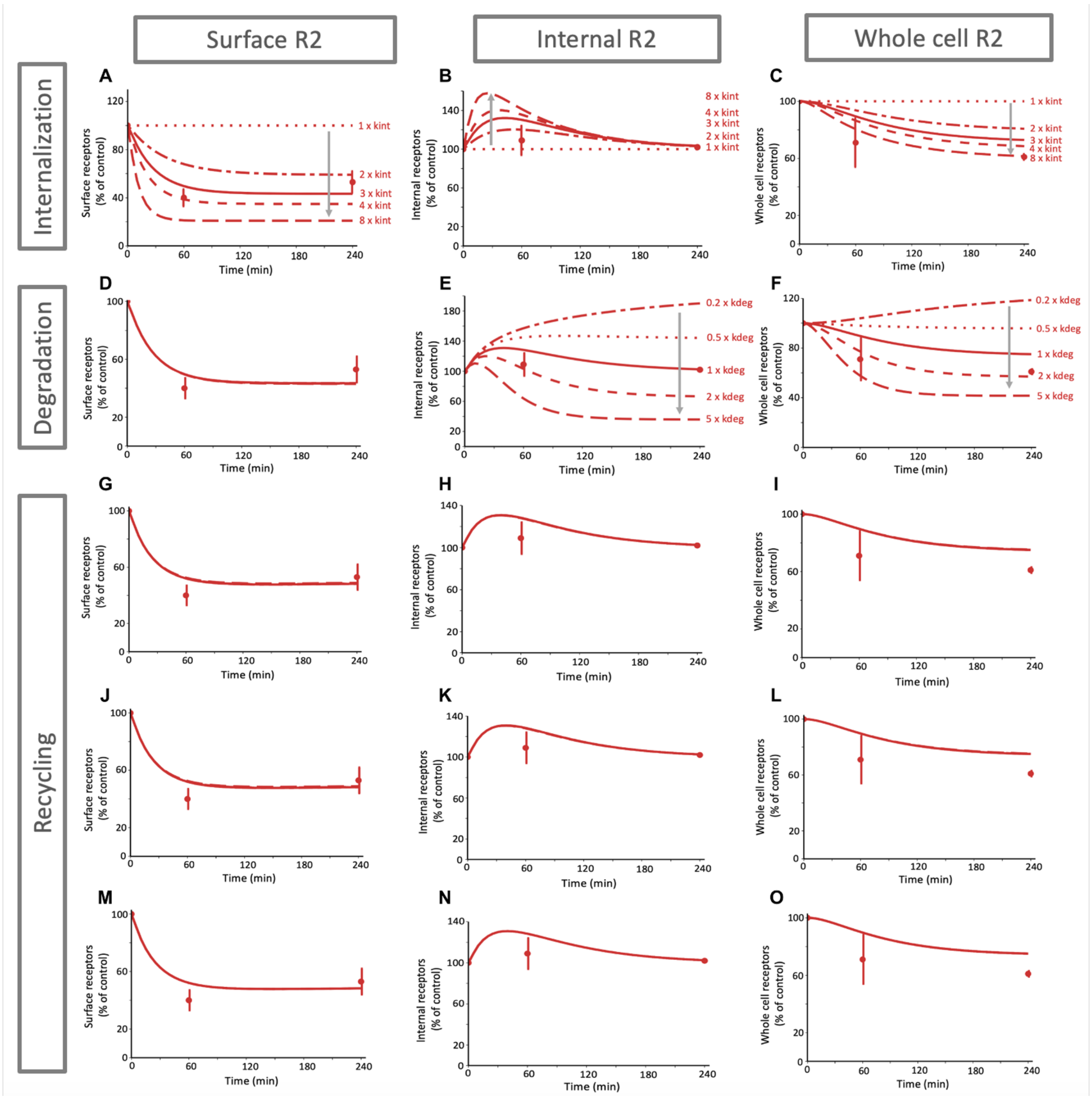

S2 Fig. Distribution of VEGFR2 over 4 hours of $VEGF_{165a}$ treatment. Total (ligated and unligated) receptor levels on the cell surface, inside the cell, and across the whole cell in response to $VEGF_{165a}$ treatment at different levels of how the ligand binding affects the indicated VEGFR2 trafficking parameter. Simulations are shown as solid and dotted lines. Gray arrows indicate the direction of increasing parameter values. The same experimental data is shown in each row as dots and variance bars. **A-C,** $VEGF_{165a}$ binding causes an increase in the internalization rate constant for VEGFR2 (V.R2.kint and V.R2.N1.kint), compared to the unligated value; a three-fold increase (solid line) compared to the unligated receptor rate constant matched the observed data best. **D-O**, Taking this increased k_{int} (receptor complex internalization rate) as a baseline for the remaining simulations, we explored variation in the other trafficking parameters. Lines represent 5x, 2x, 1x, 0.5x, 0.2x the baseline (unligated) trafficking rate; in panels D and G-O the lines overlap substantially. **D-F,** total surface, internal and whole cell receptors in response to $VEGF_{165a}$ treatment and change in ligated VEGFR2 degradation (altered k_{deg} affecting both V.R2 and V.R2.N1

complexes). **G-O,** total surface, internal and whole cell receptors in response to $VEGF_{165a}$ treatment and change in ligated VEGFR2 recycling, including: recycling via Rab4a (altered k_{rec4}, G-I); transfer to Rab11a (altered k_{4to11}, J-L); and recycling via Rab11a (altered k_{rec11}, M-O). Altered rate constants affect both V.R2 and V.R2.N1 complexes. Panels A-F are identical to panels A-F of Fig 3; they are replicated here for ease of comparison. Gray arrows indicate the direction of increasing parameter values.
