# Supplementary material for: Impact of ligand binding on VEGFR1, VEGFR2, and NRP1 localization in human endothelial cells
Source: PLoS Comput Biol. 2025 Jul 16;21(7):e1013254. doi: 10.1371/journal.pcbi.1013254 (PMC12310042; doi:10.1371/journal.pcbi.1013254)
Supplement: S4 Fig — Total (ligated and unligated) receptor levels on the cell surface, inside the cell, and across the whole cell in response to PLGF1 treatment at different levels of how the ligand binding affects the indicated VEGFR1 trafficking parameter. Simulations are shown as solid and dotted lines. Lines represent 5x, 2x, 1x, 0.5x, 0.2x the baseline (unligated) trafficking rate. Gray arrows indicate the direction of increasing parameter values. The same experimental data is shown in each row as dots and variance bars. A-C, Response to PLGF1 treatment for different values of the ligated VEGFR1 internalization parameter (kint, applies to both P.R1 and P.R1.N1 complexes), D-F, Response to PLGF1 treatment for different values of the ligated VEGFR1 degradation parameter (kdeg), G-I, Response to PLGF1 treatment for different values of the ligated VEGFR1 fast recycling via Rab4a-expressing endosomes parameter (krec4), J-L, Response to PLGF1 treatment for different values of the ligated VEGFR1 transfer from Rab4a-expressing endosomes to Rab11a-expressing endosomes parameter (k4to11), M-O, Response to PLGF1 treatment for different values of the ligated VEGFR1 slow recycling via Rab11a-expressing endosomes parameter (krec11). (PDF) [file pcbi.1013254.s024.pdf]

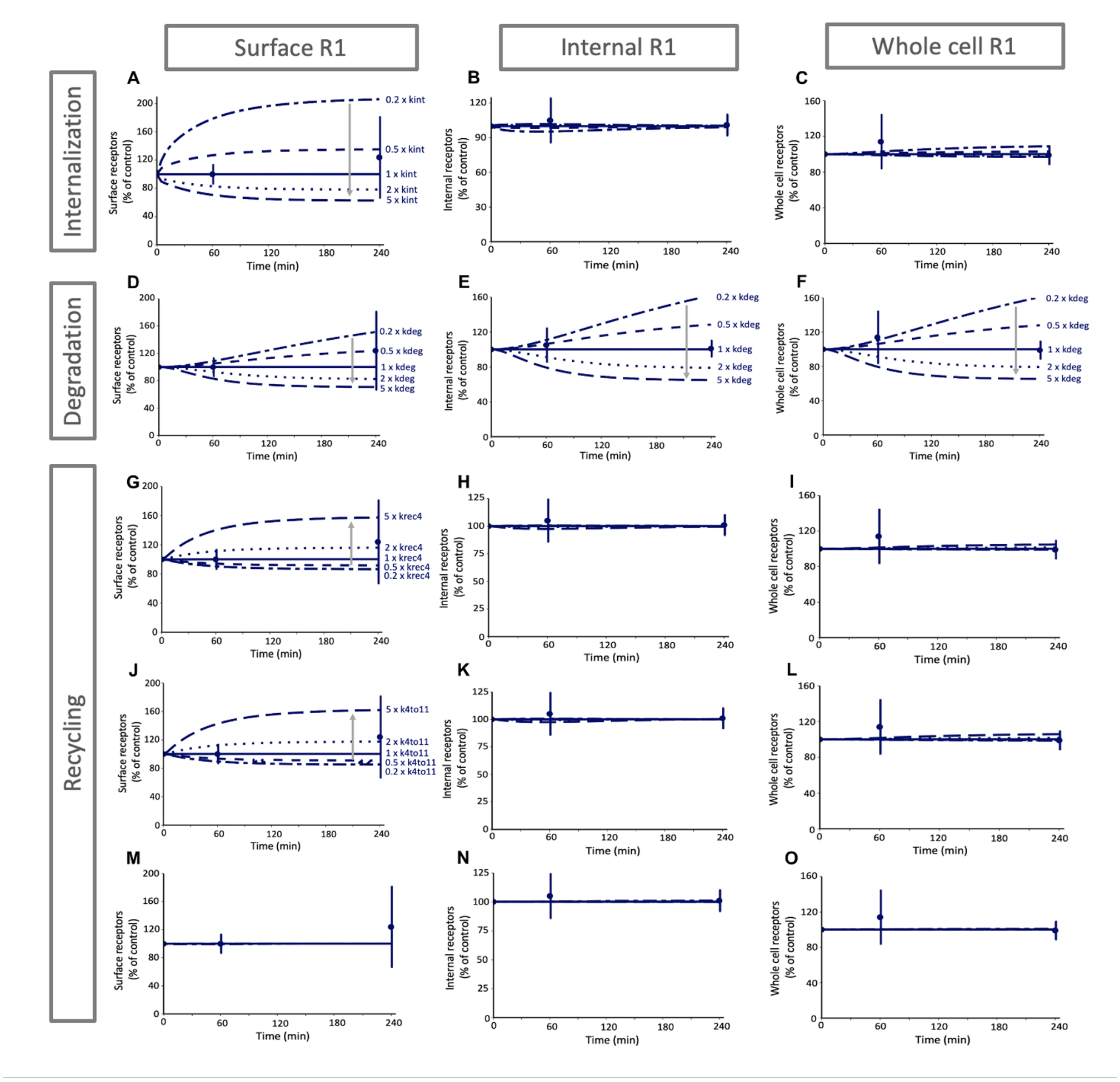

S4 Fig. Distribution of VEGFR1 over 4 hours of $PLGF_1$ treatment. Total (ligated and unligated) receptor levels on the cell surface, inside the cell, and across the whole cell in response to $PLGF_1$ treatment at different levels of how the ligand binding affects the indicated VEGFR1 trafficking parameter. Simulations are shown as solid and dotted lines. Lines represent 5x, 2x, 1x, 0.5x, 0.2x the baseline (unligated) trafficking rate. Gray arrows indicate the direction of increasing parameter values. The same experimental data is shown in each row as dots and variance bars. **A-C,** Response to PLGF1 treatment for different values of the ligated VEGFR1 internalization parameter (k_{int}, applies to both P.R1 and P.R1.N1 complexes), **D-F,** Response to $PLGF_1$ treatment for different values of the ligated VEGFR1 degradation parameter (k_{deg}), **G-I,** Response to $PLGF_1$ treatment for different values of the ligated VEGFR1 fast recycling via Rab4a-expressing endosomes parameter (k_{rec4}), **J-L,** Response to $PLGF_1$ treatment for different values of the ligated VEGFR1 transfer from Rab4a-expressing endosomes to Rab11a-expressing endosomes parameter (k_{4to11}), **M-O,** Response to $PLGF_1$ treatment for different values of the ligated VEGFR1 slow recycling via Rab11a-expressing endosomes parameter (k_{rec11}).
