# Supplementary material for: Impact of ligand binding on VEGFR1, VEGFR2, and NRP1 localization in human endothelial cells
Source: PLoS Comput Biol. 2025 Jul 16;21(7):e1013254. doi: 10.1371/journal.pcbi.1013254 (PMC12310042; doi:10.1371/journal.pcbi.1013254)
Supplement: S6 Fig — Predicted levels of VEGFR1, VEGFR2, and NRP1 at the surface, internally, and across the whole cell (“total”) after 0, 60, or 240 mins treatment with 50 ng.mL-1 of PLGF1 (A-B) or PLGF2 (C-D). Each row represents simulations with a different ligated receptor trafficking parameter increased five-fold. Changes in receptor levels normalized to the no-ligand condition (0 mins) are shown (A,C). These are further normalized to the no-parameter-change (“ctrl”) condition (B,D), demonstrating the small number of parameters that alter receptor localization. (PDF) [file pcbi.1013254.s026.pdf]

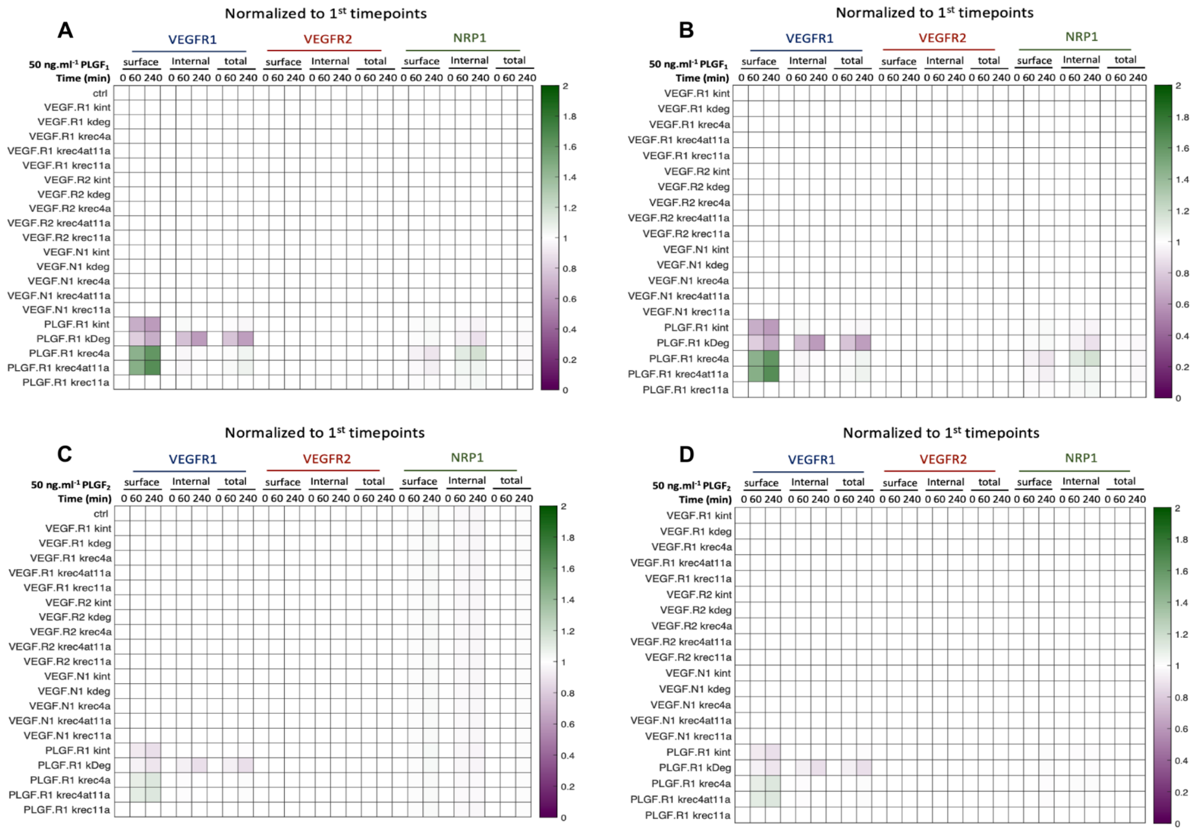

S6 Fig. Summary of impact of trafficking parameters on localization of VEGF receptors following PLGF ligation. Predicted levels of VEGFR1, VEGFR2, and NRP1 at the surface, internally, and across the whole cell ("total") after 0, 60, or 240 mins treatment with 50 ng.mL^{-1} of $PLGF_1$ (A-B) or $PLGF_2$ (C-D). Each row represents simulations with a different ligated receptor trafficking parameter increased five-fold. Changes in receptor levels normalized to the no-ligand condition (0 mins) are shown (A,C). These are further normalized to the no-parameter-change ("ctrl") condition (B,D), demonstrating the small number of parameters that alter receptor localization.
