# Supplementary material for: Impact of ligand binding on VEGFR1, VEGFR2, and NRP1 localization in human endothelial cells
Source: PLoS Comput Biol. 2025 Jul 16;21(7):e1013254. doi: 10.1371/journal.pcbi.1013254 (PMC12310042; doi:10.1371/journal.pcbi.1013254)
Supplement: S7 Fig — The overall transport rates (rate constant multiplied by concentration) for VEGFR1, VEGFR2, and NRP1 in each subcellular location at steady state (no ligand treatment) and under different durations of ligand treatment. A rate constant may be high, but if the corresponding concentration is low (and we know that the receptors are not uniformly distributed across cellular compartments), then the rate of movement will be low. Note also that the x-axis scale is different for each receptor. At steady state, these overall rates in and out are balanced, so the ‘net’ rates are close to zero. For Rab11a: receptors arriving from Rab4a are balanced out by recycling; for Rab4a: receptors internalized to Rab4a are balanced out the sum of degraded and recycling; for surface: receptor internalization balances the sum of new synthesis and recycling. “degr.” = degradation; “prodn.” = production. A-C, transport rates for VEGFR1, VEGFR2, and NRP1 at steady state starvation conditions (no ligand treatment), D-F, transport rates for VEGFR1, VEGFR2, and NRP1 after 1 hour of 50 ng.mL-1 VEGF165a treatment, G-I, transport rates for VEGFR1, VEGFR2, and NRP1 after 4 hours of 50 ng.mL-1 VEGF165a treatment. (PDF) [file pcbi.1013254.s027.pdf]

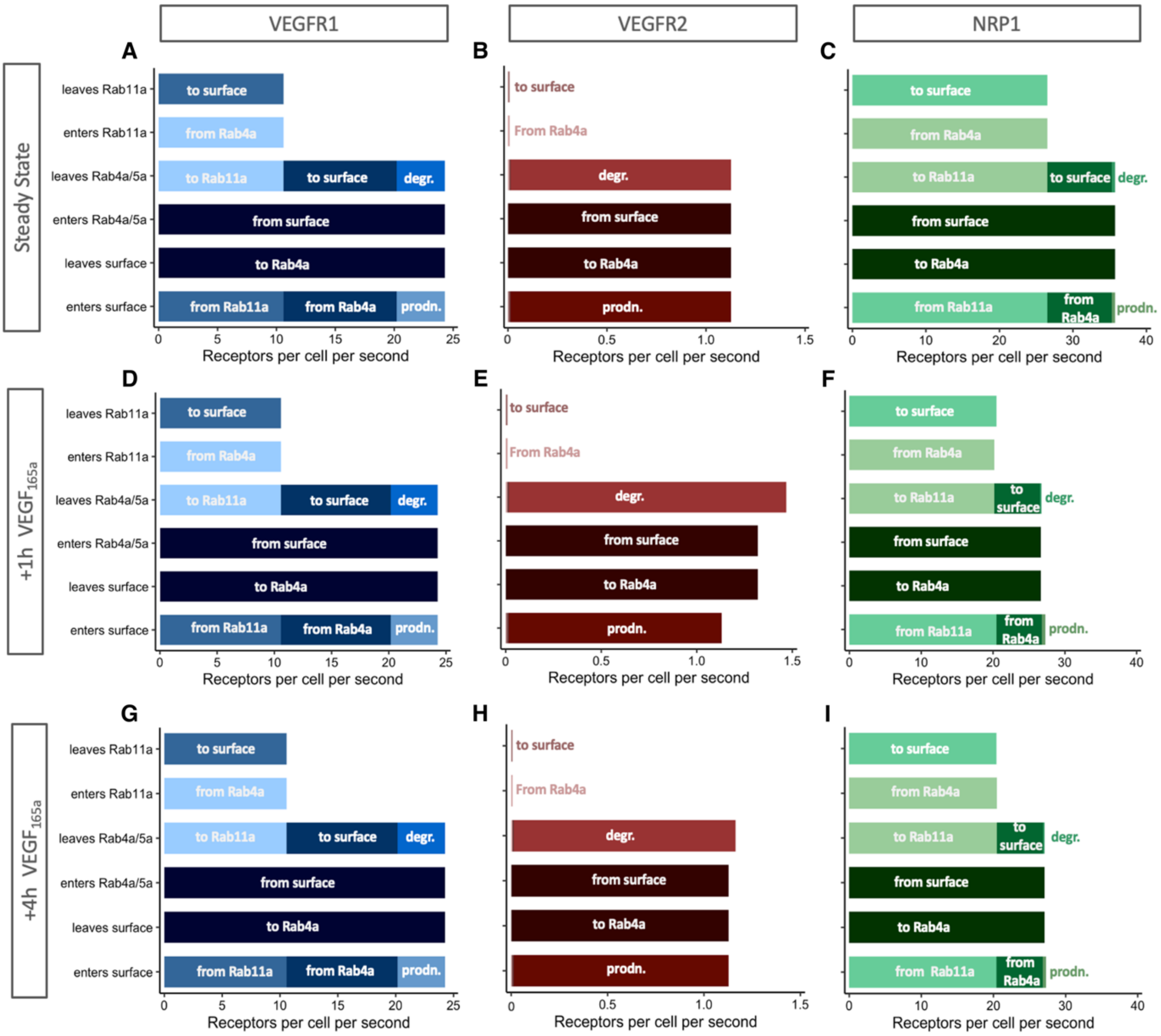

S7 Fig. Differential receptor fluxes. The overall transport rates (rate constant multiplied by concentration) for VEGFR1, VEGFR2, and NRP1 in each subcellular location at steady state (no ligand treatment) and under different durations of ligand treatment. A rate constant may be high, but if the corresponding concentration is low (and we know that the receptors are not uniformly distributed across cellular compartments), then the rate of movement will be low. Note also that the x-axis scale is different for each receptor. At steady state, these overall rates in and out are balanced, so the 'net' rates are close to zero. For Rab11a: receptors arriving from Rab4a are balanced out by recycling; for Rab4a: receptors internalized to Rab4a are balanced out the sum of degraded and recycling; for surface: receptor internalization balances the sum of new synthesis and recycling. "degr." = degradation; "prodn." = production. **A-C,** transport rates for VEGFR1, VEGFR2, and NRP1 at steady state starvation conditions (no ligand treatment), **D-F,** transport rates for VEGFR1, VEGFR2, and NRP1 after 1 hour of 50 ng.mL^{-1} VEGF$_{165a}$ treatment, **G-I,** transport rates for VEGFR1, VEGFR2, and NRP1 after 4 hours of 50 ng.mL^{-1} VEGF$_{165a}$ treatment.
