# Supplementary material for: Impact of ligand binding on VEGFR1, VEGFR2, and NRP1 localization in human endothelial cells
Source: PLoS Comput Biol. 2025 Jul 16;21(7):e1013254. doi: 10.1371/journal.pcbi.1013254 (PMC12310042; doi:10.1371/journal.pcbi.1013254)
Supplement: S8 Fig — D-F, 4 hours of HUVEC treatment with 50 ng.mL-1 of VEGF165a. G-I, 1 hour of HUVEC treatment with 50 ng.mL-1 of PLGF 1. J-L, 4 hours of HUVEC treatment with 50 ng.mL-1 of PLGF1. (PDF) [file pcbi.1013254.s028.pdf]

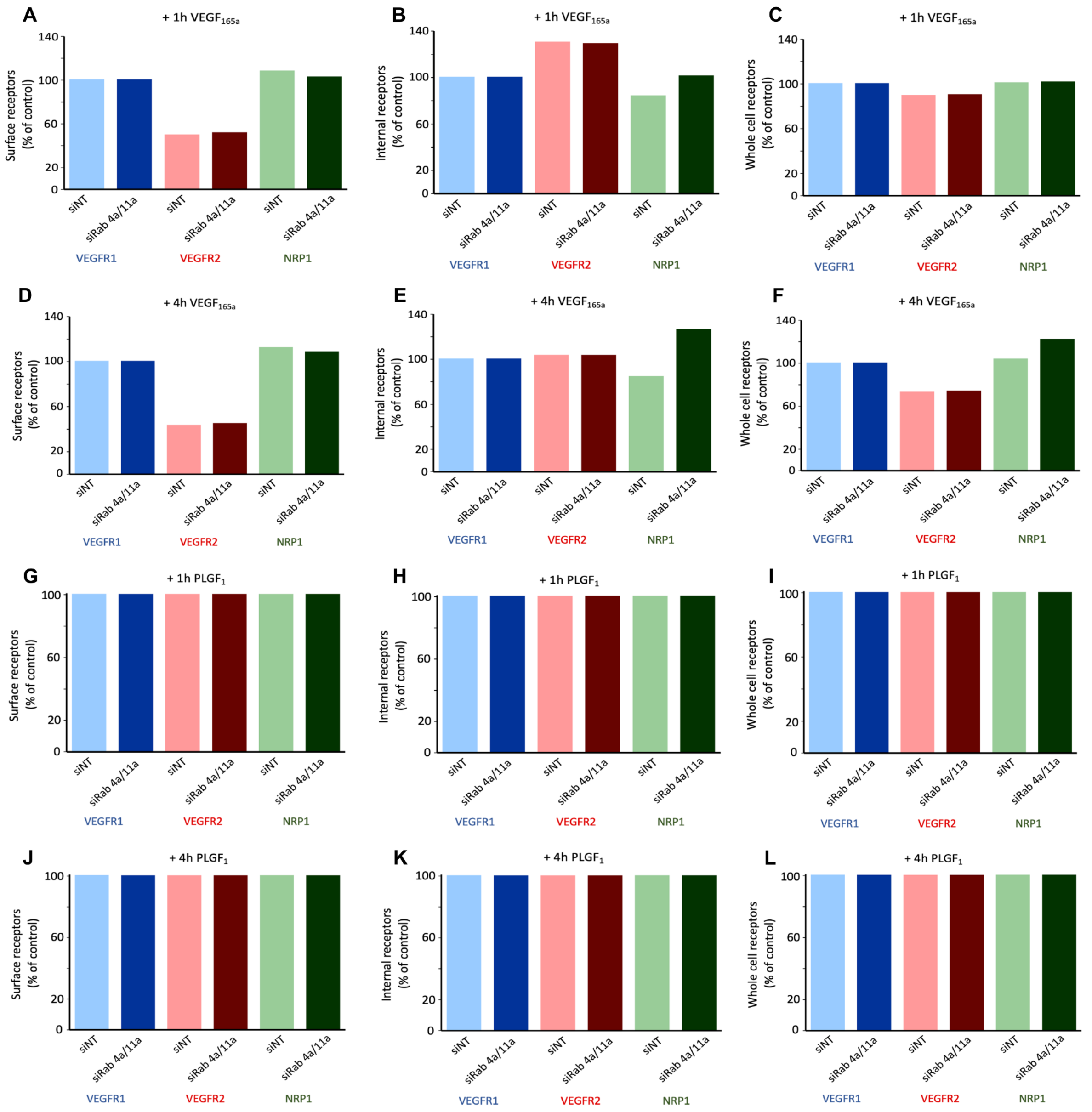


**S8 Fig.** Surface, internal and whole cell levels of total (unligated and ligated) VEGFR1, VEGFR2, and NRP1 after Rab4a/Rab11a knockdown, compared to control (no siRNA treatment) under **A-C,** 1 hour of HUVEC treatment with 50 ng.mL$^{-1}$ of VEGF$_{165a}$. **D-F,** 4 hours of HUVEC treatment with 50 ng.mL$^{-1}$ of VEGF$_{165a}$. **G-I,** 1 hour of HUVEC treatment with 50 ng.mL$^{-1}$ of PLGF $_{1}$. **J-L,** 4 hours of HUVEC treatment with 50 ng.mL$^{-1}$ of PLGF$_{1}$.
