# Supplementary material for: Impact of ligand binding on VEGFR1, VEGFR2, and NRP1 localization in human endothelial cells
Source: PLoS Comput Biol. 2025 Jul 16;21(7):e1013254. doi: 10.1371/journal.pcbi.1013254 (PMC12310042; doi:10.1371/journal.pcbi.1013254)
Supplement: S9 Fig — Previous supplemental figures S2–S7 Figs have illustrated localization of all receptors, without regard to whether they are in complexes with other receptors or ligands. Here, we focus on active, ligated receptors. Number of active (ligand-dimerized) VEGFR2 receptors on the whole cell (A-C), cell surface (D-F), and internally (G-I) following 15 min, 60 min, or 240 min of treatment with VEGF121a or VEGF165a. (PDF) [file pcbi.1013254.s029.pdf]

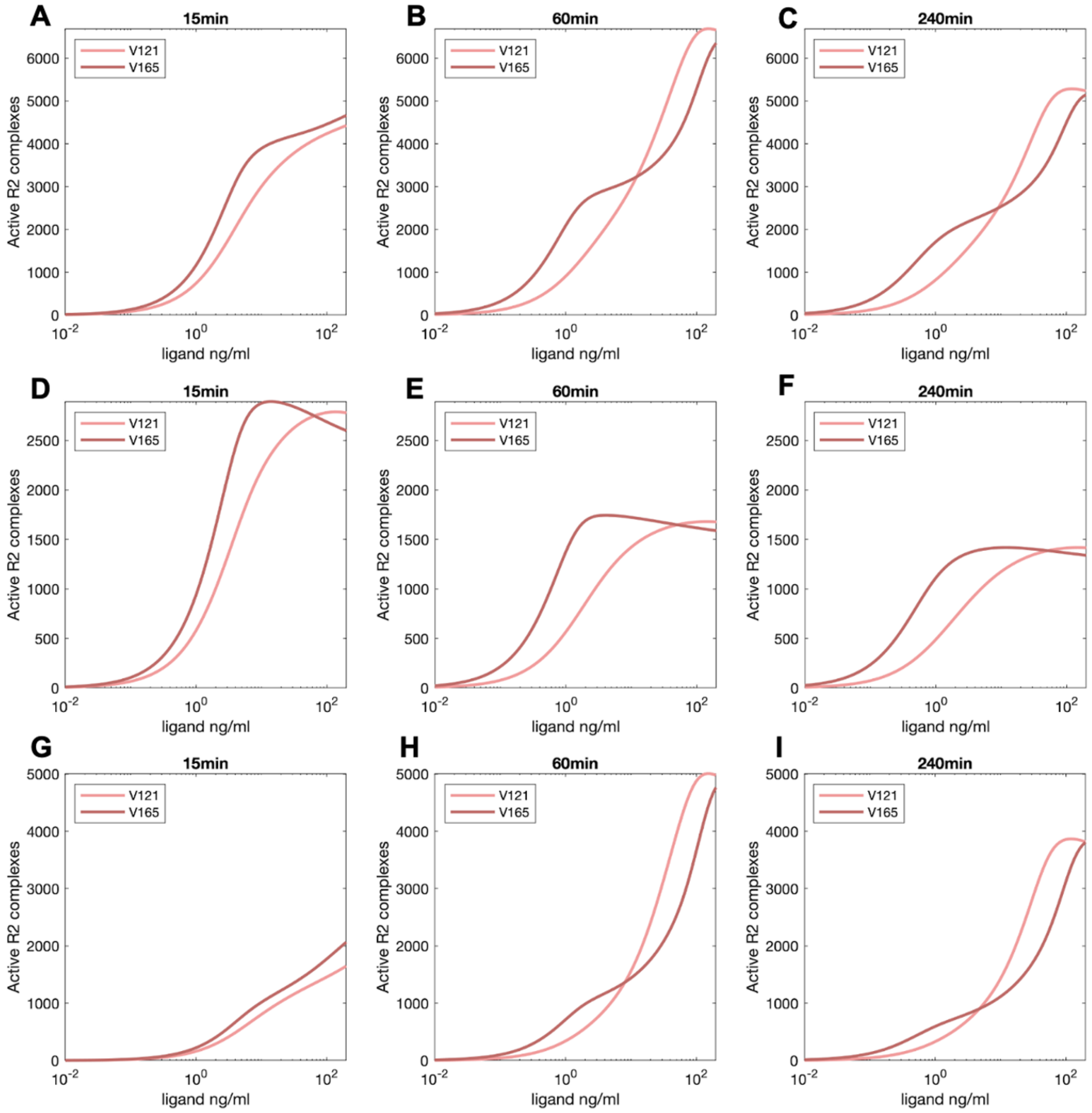

S9 Fig. Active VEGFR2 following VEGF treatment. Previous supplemental figures, S2-S7 Figs, have illustrated localization of all receptors, without regard to whether they are in complexes with other receptors or ligands. Here, we focus on active, ligated receptors. Number of active (ligand-dimerized) VEGFR2 receptors on the whole cell (A-C), cell surface (D-F), and internally (G-I) following 15 min, 60 min, or 240 min of treatment with $VEGF_{121a}$ or $VEGF_{165a}$.
