# Supplementary material for: Impact of ligand binding on VEGFR1, VEGFR2, and NRP1 localization in human endothelial cells
Source: PLoS Comput Biol. 2025 Jul 16;21(7):e1013254. doi: 10.1371/journal.pcbi.1013254 (PMC12310042; doi:10.1371/journal.pcbi.1013254)
Supplement: S10 Fig — Number of active (ligand-dimerized) VEGFR1 receptors on the whole cell (A-C), cell surface (D-F), and internally (G-I) following 15 min, 60 min, or 240 min of treatment with VEGF121a, VEGF165a, PLGF1, or PLGF2. (PDF) [file pcbi.1013254.s030.pdf]

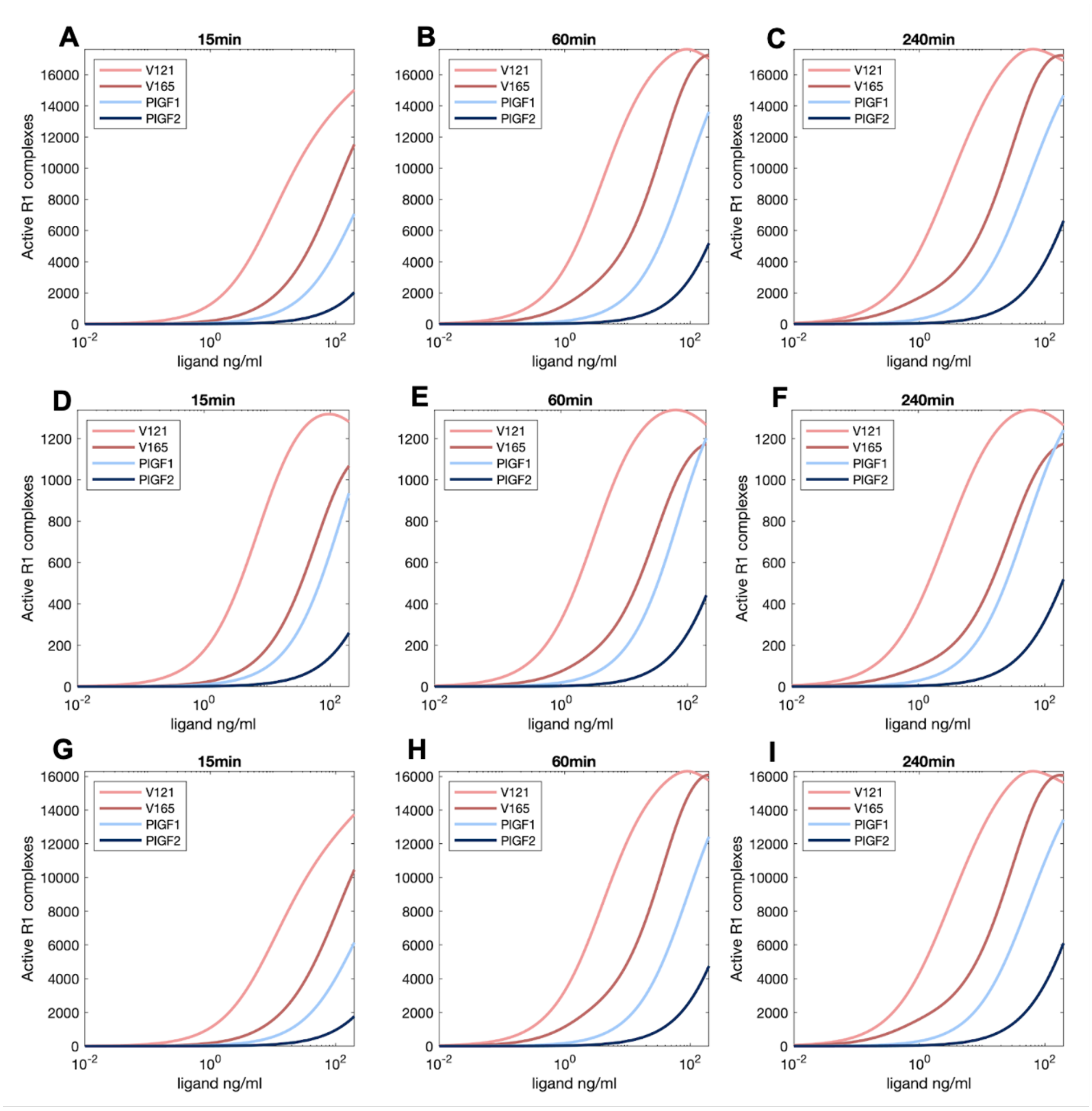

S10 Fig. Active VEGFR1 following VEGF or PLGF treatment. Number of active (ligand-dimerized) VEGFR1 receptors on the whole cell (A-C), cell surface (D-F), and internally (G-I) following 15 min, 60 min, or 240 min of treatment with $VEGF_{121a}$, $VEGF_{165a}$, $PLGF_1$, or $PLGF_2$.
