# Supplementary material for: Impact of ligand binding on VEGFR1, VEGFR2, and NRP1 localization in human endothelial cells
Source: PLoS Comput Biol. 2025 Jul 16;21(7):e1013254. doi: 10.1371/journal.pcbi.1013254 (PMC12310042; doi:10.1371/journal.pcbi.1013254)
Supplement: S11 Fig — (PDF) [file pcbi.1013254.s031.pdf]

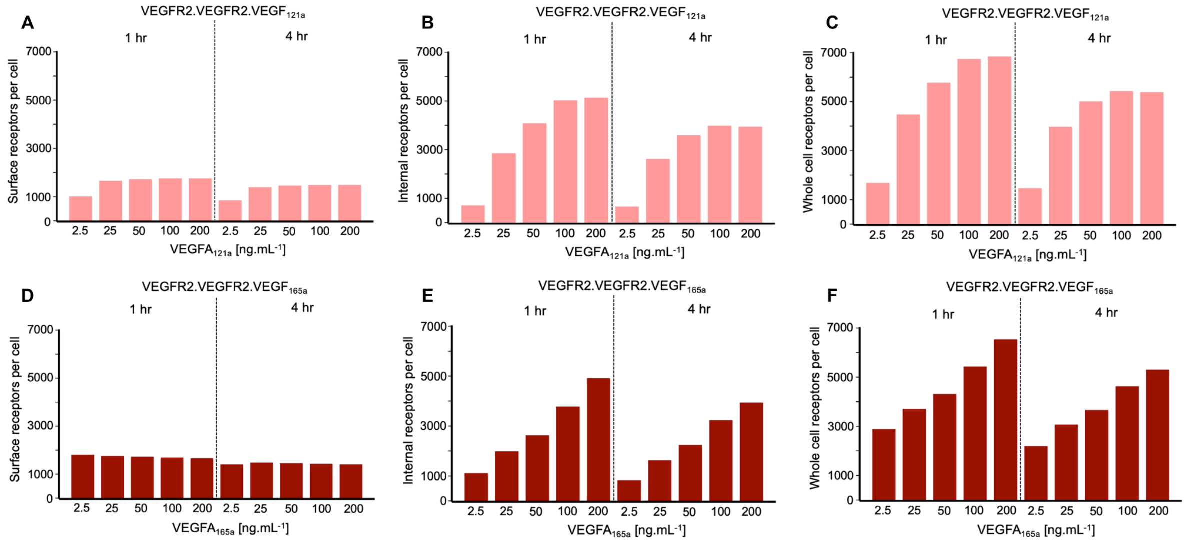

S11 Fig. Ligand dose-dependent distribution of ligated VEGFR2. different single ligand doses 2.5, 25, 50, 100, 200 ng.mL^{-1} of **A-C,** VEGF$_{121a}$ treatment or **D-F,** VEGF$_{165a}$ treatment.
