# Supplementary material for: Impact of ligand binding on VEGFR1, VEGFR2, and NRP1 localization in human endothelial cells
Source: PLoS Comput Biol. 2025 Jul 16;21(7):e1013254. doi: 10.1371/journal.pcbi.1013254 (PMC12310042; doi:10.1371/journal.pcbi.1013254)
Supplement: S13 Fig — VEGF receptors can be dimerized in the absence of ligand; they can also be ligand-bound and dimerized without being active (if the ligand is not bound to both receptors). These graphs show the impact on receptor dimerization (not activation) across the whole cell (A-C), or on the cell surface (D-F) after 240 min of treatment with 50 ng.ml-1 VEGF121a, VEGF165a, PLGF1, or PLGF2. (PDF) [file pcbi.1013254.s033.pdf]

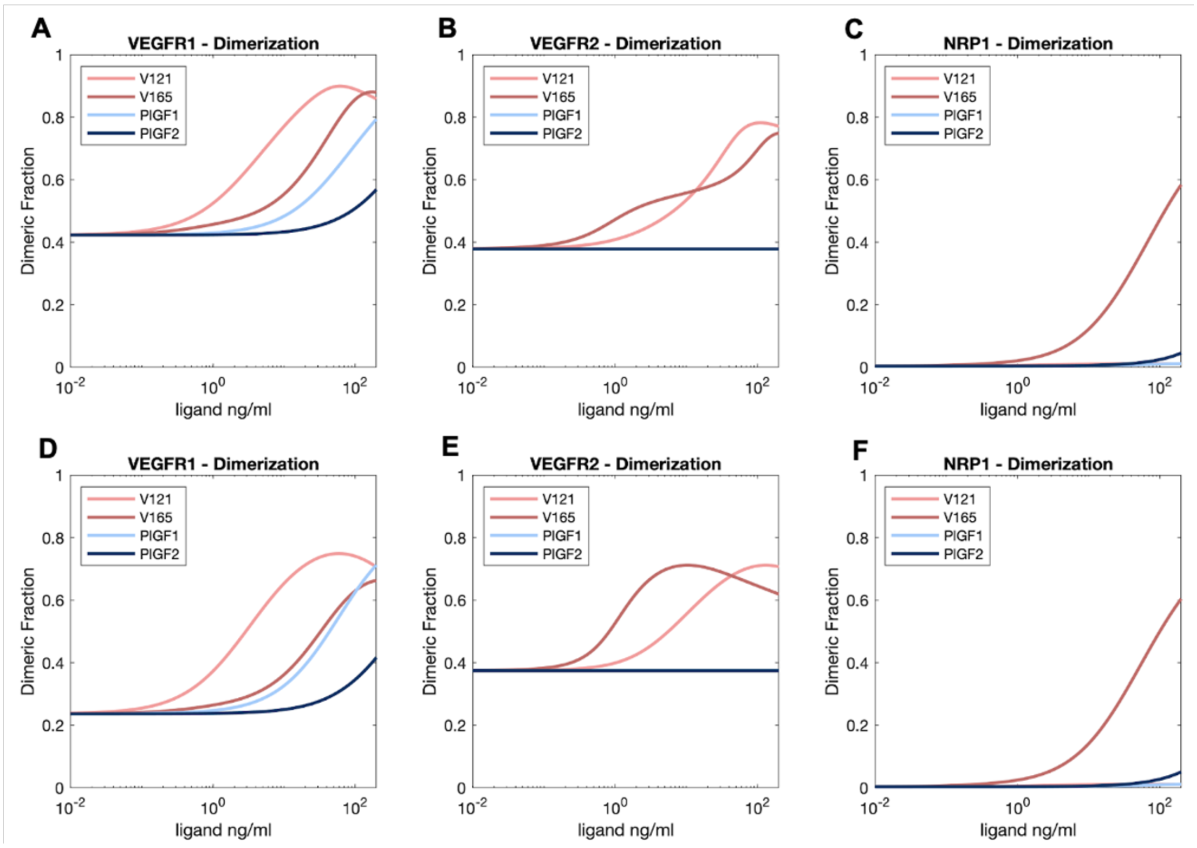

S13 Fig. Dimerization of VEGFR1, VEGFR2 and NRP1. VEGF receptors can be dimerized in the absence of ligand; they can also be ligand-bound and dimerized without being active (if the ligand is not bound to both receptors). These graphs show the impact on receptor dimerization (not activation) across the whole cell (A-C), or on the cell surface (D-F) after 240 min of treatment with 50 ng.ml^{-1} $VEGF_{121a}$, $VEGF_{165a}$, $PLGF_1$, or $PLGF_2$.
