# Supplementary material for: Impact of ligand binding on VEGFR1, VEGFR2, and NRP1 localization in human endothelial cells
Source: PLoS Comput Biol. 2025 Jul 16;21(7):e1013254. doi: 10.1371/journal.pcbi.1013254 (PMC12310042; doi:10.1371/journal.pcbi.1013254)
Supplement: S14 Fig — VEGFR1 and NRP1 can associate without ligands [45], and the VEGFR1-NRP1 complex formation affected by the presence of ligands that can bind to the complex (VEGF121a and PLGF1) and those that cannot (VEGF165a and PLGF2). These graphs show the impact of VEGFR1-NRP1 dimerization across the whole cell (A-B) or on the cell surface (C-D) after 240 minutes of 50 ng.ml-1 VEGF121a, VEGF165a, PLGF1, or PLGF2. (PDF) [file pcbi.1013254.s034.pdf]

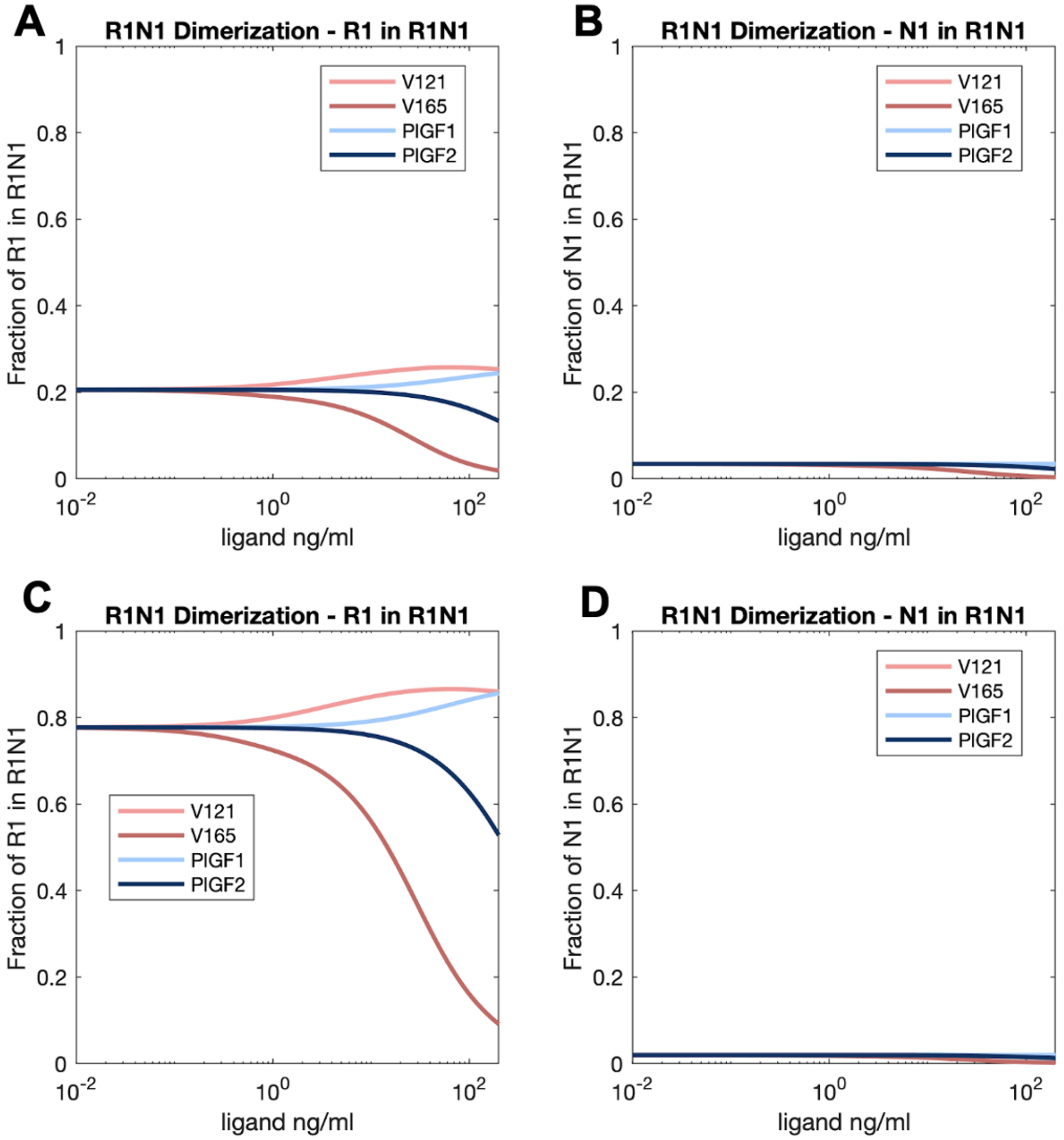

S14 Fig. Fraction of VEGFR1-NRP1 heterodimers. VEGFR1 and NRP1 can associate without ligands [45], and the VEGFR1-NRP1 complex formation affected by the presence of ligands that can bind to the complex ($VEGF_{121a}$ and $PLGF_1$) and those that cannot ($VEGF_{165a}$ and $PLGF_2$). These graphs show the impact of VEGFR1-NRP1 dimerization across the whole cell (A-B) or on the cell surface (C-D) after 240 minutes of 50 $ng.ml^{-1}$ $VEGF_{121a}$, $VEGF_{165a}$, $PLGF_1$, or $PLGF_2$.
