# Supplementary material for: Impact of ligand binding on VEGFR1, VEGFR2, and NRP1 localization in human endothelial cells
Source: PLoS Comput Biol. 2025 Jul 16;21(7):e1013254. doi: 10.1371/journal.pcbi.1013254 (PMC12310042; doi:10.1371/journal.pcbi.1013254)
Supplement: S15 Fig — A, VEGFR2.VEGF165a.VEGFR2 levels for different initial VEGF165a concentrations. B, VEGFR2.VEGF165a.VEGFR2 levels in the absence of NRP1 for different initial VEGF165a concentrations. C, percent change in VEGFR2.VEGF165a.VEGFR2 levels due to loss of NRP1 for different initial VEGF165a concentrations. (PDF) [file pcbi.1013254.s035.pdf]

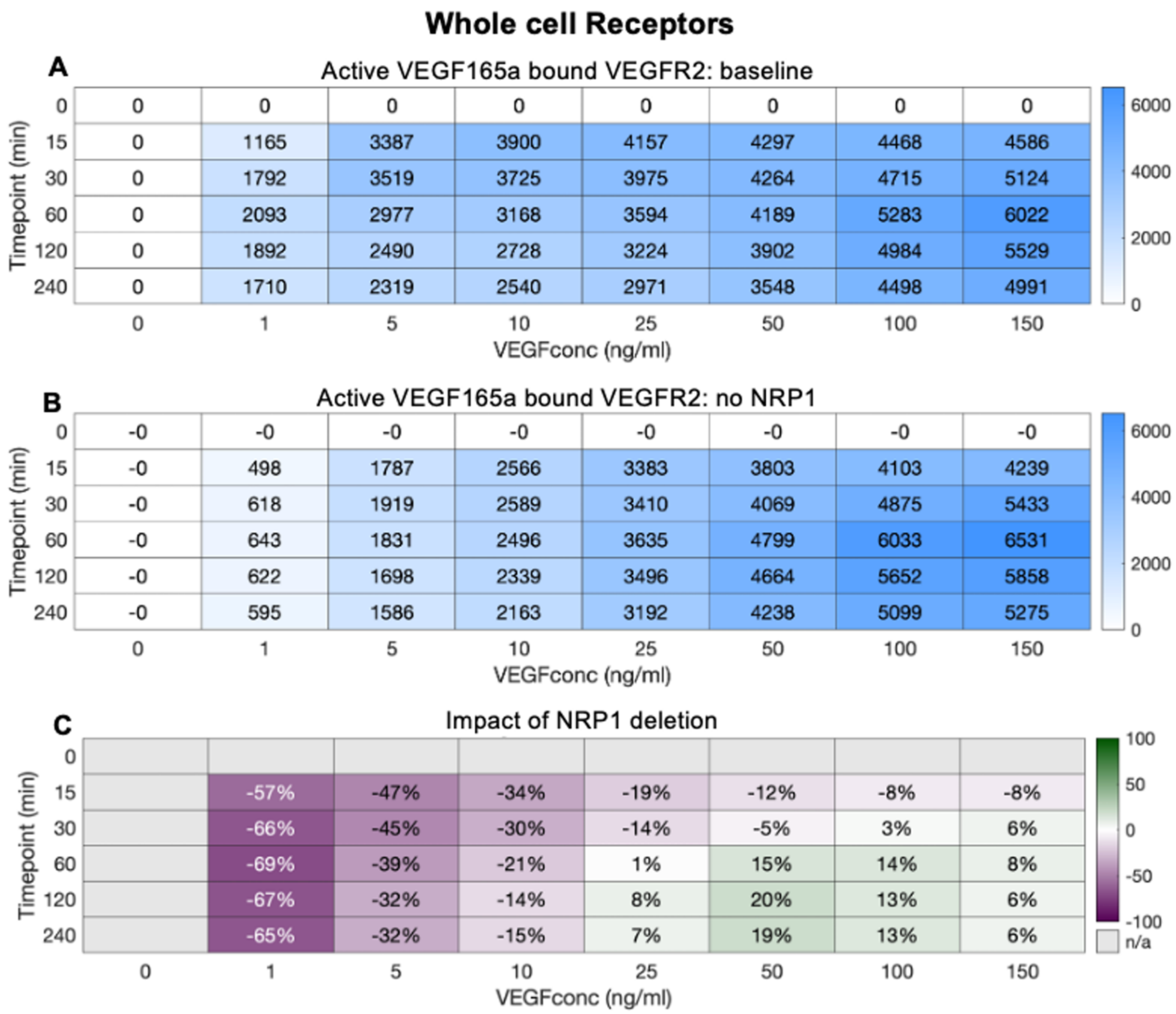

S15 Fig. Impact of NRP1 expression on whole cell VEGFR2 activation by $VEGF_{165a}$. A, $VEGFR2.VEGF_{165a}.VEGFR2$ levels for different initial $VEGF_{165a}$ concentrations. **B,** $VEGFR2.VEGF_{165a}.VEGFR2$ levels in the absence of NRP1 for different initial $VEGF_{165a}$ concentrations. **C,** percent change in $VEGFR2.VEGF_{165a}.VEGFR2$ levels due to loss of NRP1 for different initial $VEGF_{165a}$ concentrations.
