# Supplementary material for: Impact of ligand binding on VEGFR1, VEGFR2, and NRP1 localization in human endothelial cells
Source: PLoS Comput Biol. 2025 Jul 16;21(7):e1013254. doi: 10.1371/journal.pcbi.1013254 (PMC12310042; doi:10.1371/journal.pcbi.1013254)
Supplement: S16 Fig — A, VEGFR2.VEGF165a.VEGFR2 levels for different initial VEGF165a concentrations. B, VEGFR2.VEGF165a.VEGFR2 levels in the absence of NRP1 for different initial VEGF165a concentrations. C, percent change in VEGFR2.VEGF165a.VEGFR2 levels due to loss of NRP1 for different initial VEGF165a concentrations. (PDF) [file pcbi.1013254.s036.pdf]

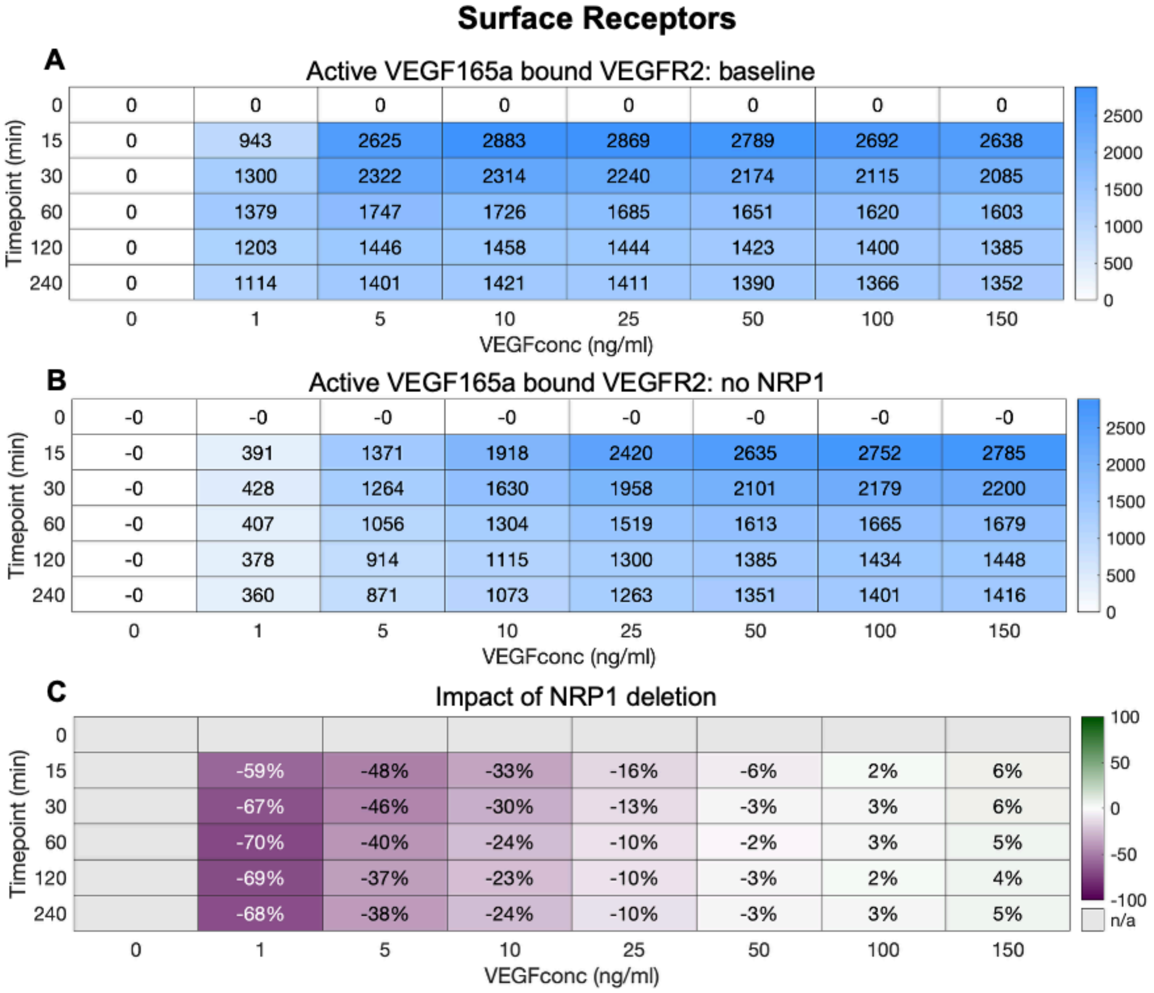

S16 Fig. Impact of NRP1 expression on surface VEGFR2 activation by VEGF$_{165a}$. A, VEGFR2.VEGF$_{165a}$.VEGFR2 levels for different initial VEGF$_{165a}$ concentrations. **B,** VEGFR2.VEGF$_{165a}$.VEGFR2 levels in the absence of NRP1 for different initial VEGF$_{165a}$ concentrations. **C,** percent change in VEGFR2.VEGF$_{165a}$.VEGFR2 levels due to loss of NRP1 for different initial VEGF$_{165a}$ concentrations.
