# Supplementary material for: Impact of ligand binding on VEGFR1, VEGFR2, and NRP1 localization in human endothelial cells
Source: PLoS Comput Biol. 2025 Jul 16;21(7):e1013254. doi: 10.1371/journal.pcbi.1013254 (PMC12310042; doi:10.1371/journal.pcbi.1013254)
Supplement: S21 Fig — A, VEGFR1.VEGF165a.VEGFR1 levels for different initial VEGF165a concentrations. B, VEGFR1.VEGF165a.VEGFR1 levels in the absence of VEGFR2 for different initial VEGF165a concentrations. C, percent change in VEGFR1.VEGF165a.VEGFR1 levels due to loss of VEGFR2 for different initial VEGF165a concentrations. (PDF) [file pcbi.1013254.s041.pdf]

Whole cell Receptors

A Active VEGFA-165a bound VEGFR1: baseline

Timepoint (min) \ VEGFconc (ng/ml)	0	1	5	10	25	50	100	150
0	0	0	0	0	0	0	0	0
15	0	206	950	1735	3596	5836	8663	10375
30	0	541	2079	3426	6467	9885	13595	15378
60	0	1225	3480	5296	9262	13088	16128	17039
120	0	1731	4043	6094	10349	14007	16568	17173
240	0	1702	3992	6082	10339	13980	16558	17167

B Active VEGFA-165a bound VEGFR1: no VEGFR2

Timepoint (min) \ VEGFconc (ng/ml)	0	1	5	10	25	50	100	150
0	-0	-0	-0	-0	-0	-0	-0	-0
15	-0	152	731	1399	3104	5244	8024	9747
30	-0	257	1239	2366	5194	8558	12454	14555
60	-0	381	1827	3460	7351	11464	15421	16951
120	-0	469	2224	4151	8447	12539	16075	17138
240	-0	492	2313	4278	8556	12580	16074	17130

C Impact of VEGFR2 deletion

Timepoint (min) \ VEGFconc (ng/ml)	0	1	5	10	25	50	100	150
0								
15		-26%	-23%	-19%	-14%	-10%	-7%	-6%
30		-52%	-40%	-31%	-20%	-13%	-8%	-5%
60		-69%	-47%	-35%	-21%	-12%	-4%	-1%
120		-73%	-45%	-32%	-18%	-10%	-3%	-0%
240		-71%	-42%	-30%	-17%	-10%	-3%	-0%

(Colour scale A, B: 0–15000; C: -100 to 100, n/a)

S21 Fig. Impact of VEGFR2 expression on whole cell VEGFR1 activation by $VEGF_{165a}$. **A,** $VEGFR1.VEGF_{165a}.VEGFR1$ levels for different initial $VEGF_{165a}$ concentrations. **B,** $VEGFR1.VEGF_{165a}.VEGFR1$ levels in the absence of VEGFR2 for different initial $VEGF_{165a}$ concentrations. **C,** percent change in $VEGFR1.VEGF_{165a}.VEGFR1$ levels due to loss of VEGFR2 for different initial $VEGF_{165a}$ concentrations.
