# Supplementary material for: Impact of ligand binding on VEGFR1, VEGFR2, and NRP1 localization in human endothelial cells
Source: PLoS Comput Biol. 2025 Jul 16;21(7):e1013254. doi: 10.1371/journal.pcbi.1013254 (PMC12310042; doi:10.1371/journal.pcbi.1013254)
Supplement: S22 Fig — A, VEGFR1.VEGF165a.VEGFR1 levels for different initial VEGF165a concentrations. B, VEGFR1.VEGF165a.VEGFR1 levels in the absence of VEGFR2 for different initial VEGF165a concentrations. C, percent change in VEGFR1.VEGF165a.VEGFR1 levels due to VEGFR2 for different initial VEGF165a concentrations. (PDF) [file pcbi.1013254.s042.pdf]

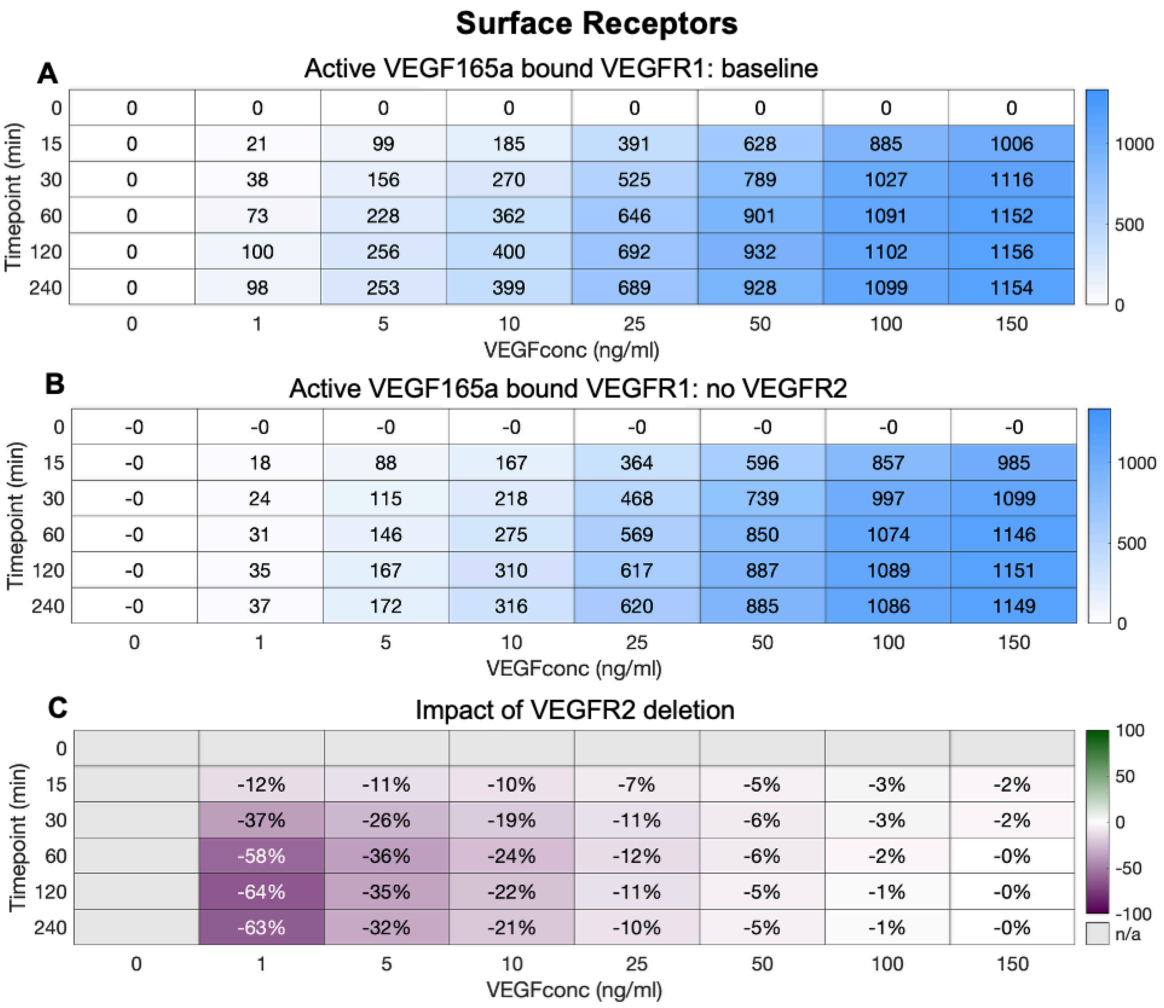

S22 Fig. Impact of VEGFR2 expression on surface VEGFR1 activation by VEGF$_{165a}$. A, VEGFR1.VEGF$_{165a}$.VEGFR1 levels for different initial VEGF$_{165a}$ concentrations. **B,** VEGFR1.VEGF$_{165a}$.VEGFR1 levels in the absence of VEGFR2 for different initial VEGF$_{165a}$ concentrations. **C,** percent change in VEGFR1.VEGF$_{165a}$.VEGFR1 levels due to VEGFR2 for different initial VEGF$_{165a}$ concentrations.
