# Supplementary material for: Impact of ligand binding on VEGFR1, VEGFR2, and NRP1 localization in human endothelial cells
Source: PLoS Comput Biol. 2025 Jul 16;21(7):e1013254. doi: 10.1371/journal.pcbi.1013254 (PMC12310042; doi:10.1371/journal.pcbi.1013254)
Supplement: S24 Fig — A, Surface, B, Internal, and C, Whole cell levels of VEGFR1.PLGF1.VEGFR1 complexes at different timepoints over 4 hours and under varying PLGF1 concentration. (PDF) [file pcbi.1013254.s044.pdf]

Active PLGF1 bound VEGFR1

A **Surface Receptors**

Timepoint (min) \ PlGFconc (ng/ml)	0	1	5	10	25	50	100	150
0	0	0	0	0	0	0	0	0
15	0	10	49	95	221	394	645	815
30	0	14	68	131	298	514	799	973
60	0	20	95	181	396	652	948	1106
120	0	26	122	229	479	746	1022	1158
240	0	29	136	252	509	770	1032	1163

B **Internal Receptors**

Timepoint (min) \ PlGFconc (ng/ml)	0	1	5	10	25	50	100	150
0	0	0	0	0	0	0	0	0
15	0	57	282	553	1301	2370	4016	5222
30	0	107	525	1020	2351	4152	6704	8414
60	0	180	868	1665	3704	6230	9377	11223
120	0	255	1213	2285	4841	7668	10736	12356
240	0	298	1397	2586	5271	8046	10941	12469

C **Whole cell Receptors**

Timepoint (min) \ PlGFconc (ng/ml)	0	1	5	10	25	50	100	150
0	0	0	0	0	0	0	0	0
15	0	67	331	648	1522	2764	4661	6037
30	0	121	593	1152	2649	4666	7504	9387
60	0	199	963	1846	4100	6882	10325	12329
120	0	281	1335	2513	5320	8414	11758	13514
240	0	328	1533	2837	5780	8817	11974	13633

S24 Fig. Induction of active VEGFR1 by PLGF$_1$. A, Surface, **B,** Internal, and **C,** Whole cell levels of VEGFR1.PLGF1.VEGFR1 complexes at different timepoints over 4 hours and under varying PLGF1 concentration.
