# Supplementary material for: Impact of ligand binding on VEGFR1, VEGFR2, and NRP1 localization in human endothelial cells
Source: PLoS Comput Biol. 2025 Jul 16;21(7):e1013254. doi: 10.1371/journal.pcbi.1013254 (PMC12310042; doi:10.1371/journal.pcbi.1013254)
Supplement: S26 Fig — Predicted level of active VEGFR1.PLGF.VEGFR1 complexes, following two hours of ligand treatment at different doses for PLGF1 and VEGF165, across the whole cell (top), on the cell surface (middle), and intracellularly (bottom). (PDF) [file pcbi.1013254.s046.pdf]

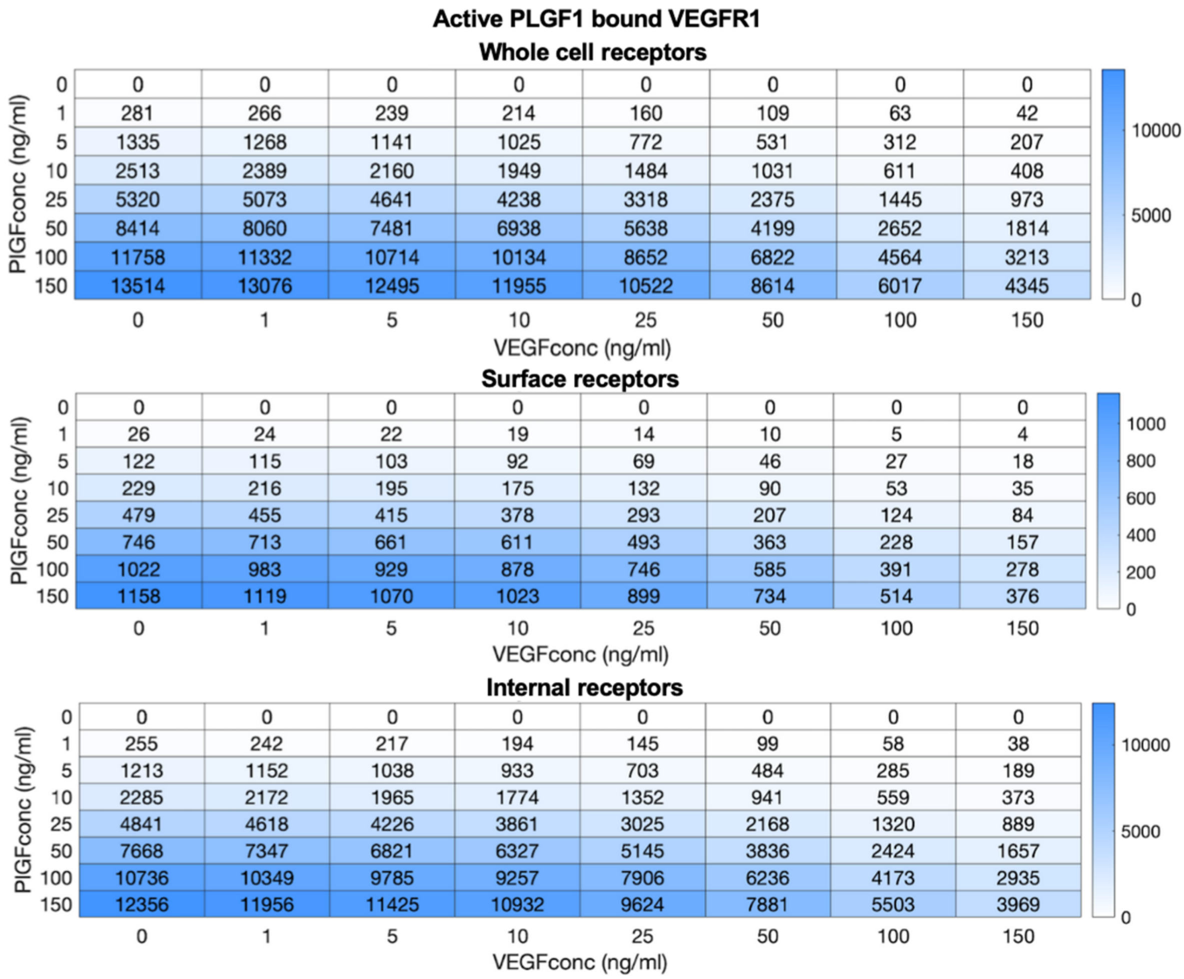

S26 Fig. PLGF-VEGF competition: impact on active PLGF-bound VEGFR1 complexes. Predicted level of active VEGFR1.PLGF.VEGFR1 complexes, following two hours of ligand treatment at different doses for $PLGF_1$ and $VEGF_{165}$, across the whole cell (top), on the cell surface (middle), and intracellularly (bottom).
