# Supplementary material for: Impact of ligand binding on VEGFR1, VEGFR2, and NRP1 localization in human endothelial cells
Source: PLoS Comput Biol. 2025 Jul 16;21(7):e1013254. doi: 10.1371/journal.pcbi.1013254 (PMC12310042; doi:10.1371/journal.pcbi.1013254)
Supplement: S27 Fig — Predicted level of free (unbound) VEGF165 (top) or PLGF1 (bottom), at the cell surface or intracellularly, following two hours of ligand treatment at different doses for PLGF1 and VEGF165. Ligand levels are given in units of #/cell. (PDF) [file pcbi.1013254.s047.pdf]

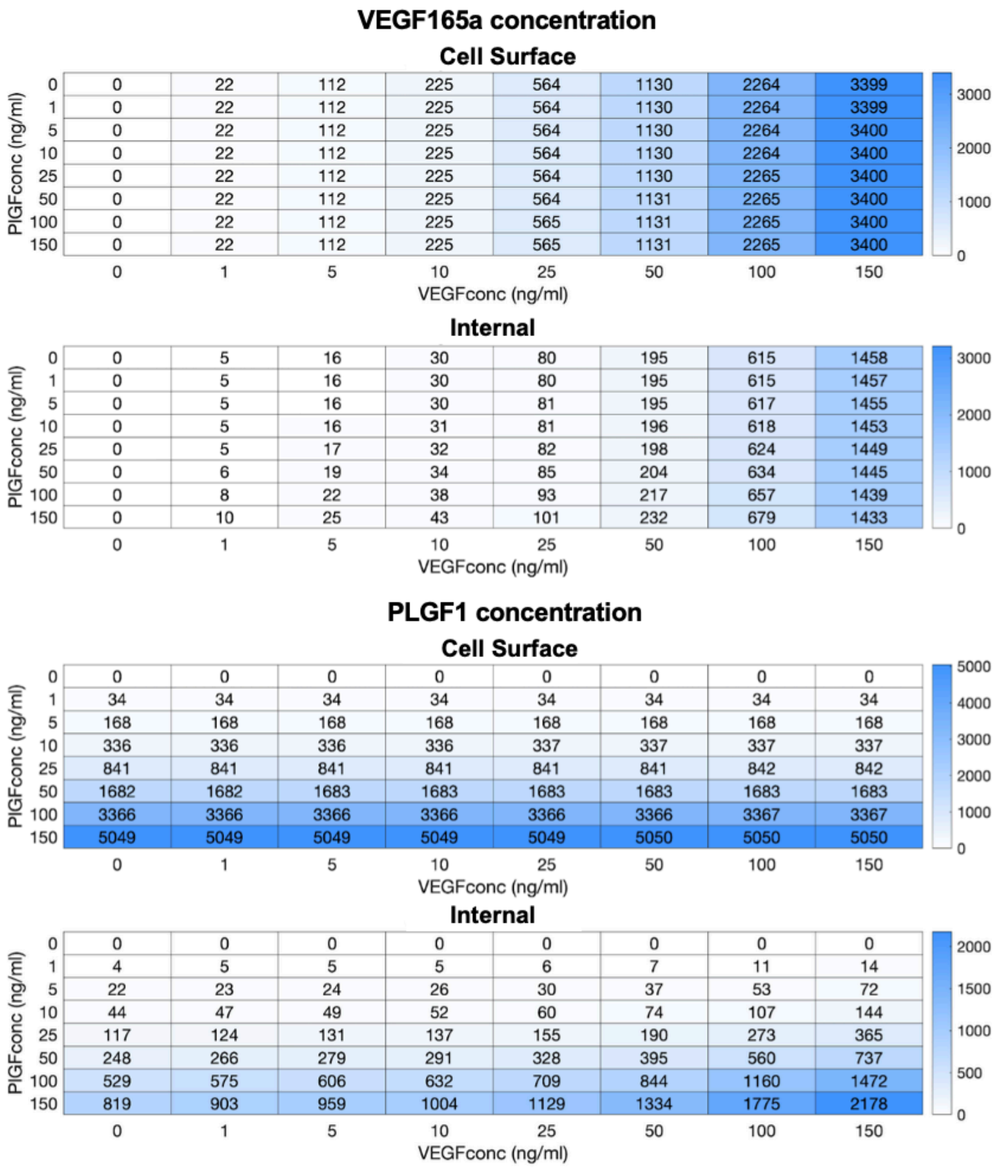

S27 Fig. PLGF-VEGF competition: ligand levels. Predicted level of free (unbound) $VEGF_{165}$ (top) or $PLGF_1$ (bottom), at the cell surface or intracellularly, following two hours of ligand treatment at different doses for $PLGF_1$ and $VEGF_{165}$. Ligand levels are given in units of #/cell.
