# Supplementary material for: Impact of ligand binding on VEGFR1, VEGFR2, and NRP1 localization in human endothelial cells
Source: PLoS Comput Biol. 2025 Jul 16;21(7):e1013254. doi: 10.1371/journal.pcbi.1013254 (PMC12310042; doi:10.1371/journal.pcbi.1013254)
Supplement: S28 Fig — Predicted level of active VEGFR2.VEGF.VEGFR2 complexes, following two hours of ligand treatment at different doses for PLGF1 and VEGF165, across the whole cell (top), on the cell surface (middle), and intracellularly (bottom). (PDF) [file pcbi.1013254.s048.pdf]

Active VEGF165a bound VEGFR2

Whole cell receptors

PlGFconc (ng/ml) \ VEGFconc (ng/ml)	0	1	5	10	25	50	100	150
0	0	1892	2490	2728	3224	3902	4984	5529
1	0	1893	2491	2730	3227	3905	4985	5529
5	0	1898	2498	2738	3239	3920	4992	5529
10	0	1903	2507	2749	3254	3937	5001	5530
25	0	1922	2535	2782	3299	3989	5026	5534
50	0	1953	2581	2838	3373	4070	5065	5539
100	0	2016	2677	2951	3515	4214	5128	5548
150	0	2080	2772	3061	3647	4335	5175	5554

Surface receptors

PlGFconc (ng/ml) \ VEGFconc (ng/ml)	0	1	5	10	25	50	100	150
0	0	1203	1446	1458	1444	1423	1400	1385
1	0	1203	1446	1458	1444	1423	1400	1385
5	0	1204	1446	1457	1444	1423	1400	1386
10	0	1204	1446	1457	1444	1424	1400	1386
25	0	1205	1446	1457	1445	1424	1400	1386
50	0	1206	1446	1457	1445	1425	1401	1386
100	0	1207	1445	1457	1445	1426	1402	1387
150	0	1209	1445	1457	1446	1426	1403	1388

Internal receptors

PlGFconc (ng/ml) \ VEGFconc (ng/ml)	0	1	5	10	25	50	100	150
0	0	688	1044	1270	1780	2479	3584	4143
1	0	689	1045	1272	1783	2482	3586	4143
5	0	694	1052	1281	1795	2496	3593	4144
10	0	700	1061	1291	1810	2514	3601	4145
25	0	717	1089	1324	1854	2565	3626	4148
50	0	747	1136	1381	1928	2645	3664	4153
100	0	809	1232	1493	2070	2789	3726	4161
150	0	870	1327	1604	2201	2909	3772	4166

S28 Fig. PLGF-VEGF competition: impact on active VEGF-bound VEGFR2 complexes. Predicted level of active VEGFR2.VEGF.VEGFR2 complexes, following two hours of ligand treatment at different doses for $PLGF_1$ and $VEGF_{165}$, across the whole cell (top), on the cell surface (middle), and intracellularly (bottom).
