# Supplementary material for: Impact of ligand binding on VEGFR1, VEGFR2, and NRP1 localization in human endothelial cells
Source: PLoS Comput Biol. 2025 Jul 16;21(7):e1013254. doi: 10.1371/journal.pcbi.1013254 (PMC12310042; doi:10.1371/journal.pcbi.1013254)
Supplement: S29 Fig — Simulations with slower (top) or faster (bottom) rate constants in the endosomes than on the cell surface, due to pH differences. Effect of 4 hours of 50 ng.mL-1 VEGF165a treatment on the intracellular levels of VEGFR2.VEGF165a.VEGFR2 in HUVECs. (PDF) [file pcbi.1013254.s049.pdf]

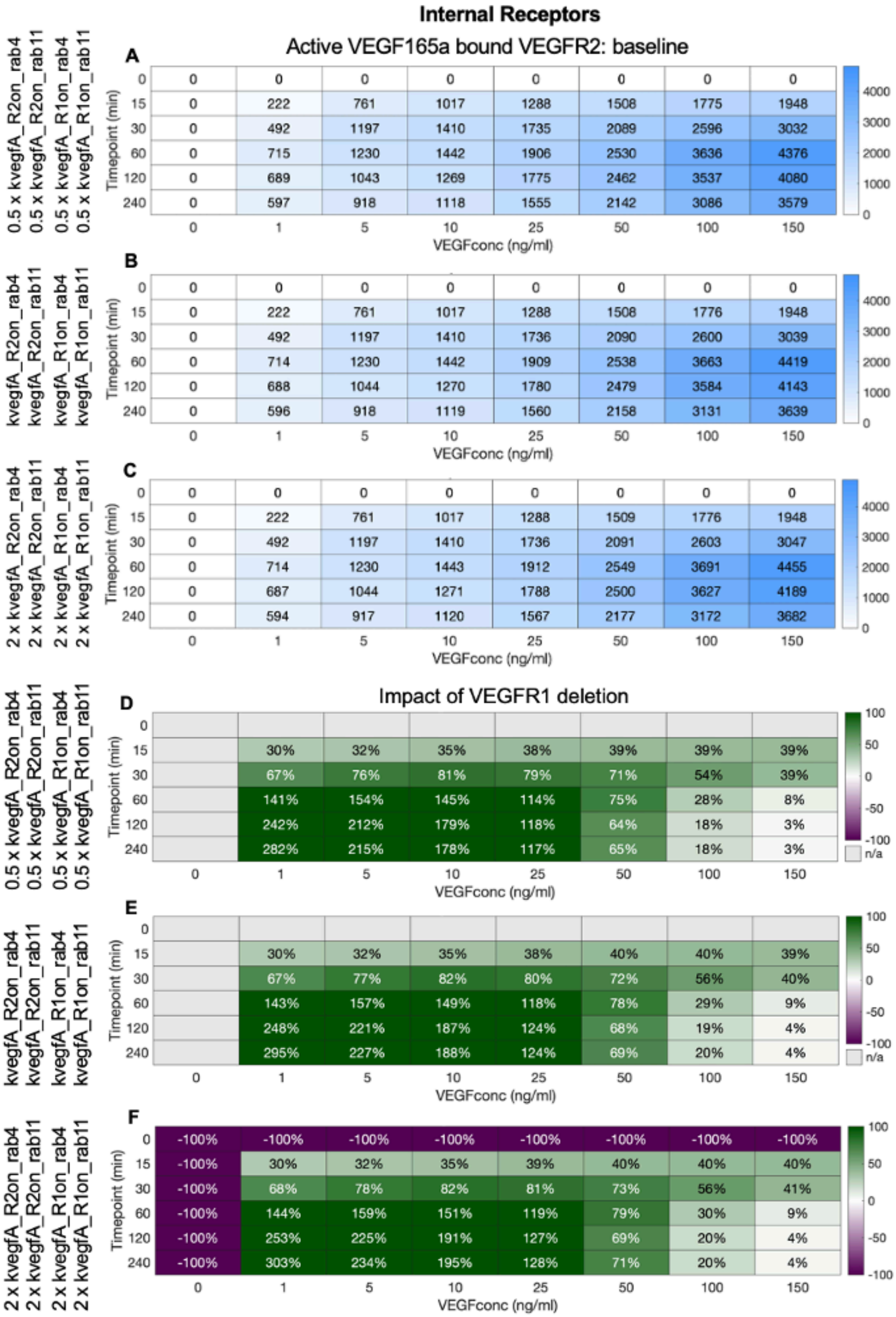

S29 Fig. Effect of change in endosomal pH on VEGFR2 activation and decoy effect. Simulations with slower (A, D) or faster (C, F) rate constants in the endosomes than on the cell surface, due to pH differences. Effect of 4 hours of 50 ng.mL^{-1} VEGF$_{165a}$ treatment on the intracellular levels of VEGFR2.VEGF$_{165a}$.VEGFR2 in HUVECs.
